# A gene catalogue of the Sprague-Dawley rat gut metagenome

**DOI:** 10.1093/gigascience/giy055

**Published:** 2018-05-11

**Authors:** Hudan Pan, Ruijin Guo, Jie Zhu, Qi Wang, Yanmei Ju, Ying Xie, Yanfang Zheng, Zhifeng Wang, Ting Li, Zhongqiu Liu, Linlin Lu, Fei Li, Bin Tong, Liang Xiao, Xun Xu, Runze Li, Zhongwen Yuan, Huanming Yang, Jian Wang, Karsten Kristiansen, Huijue Jia, Liang Liu

**Affiliations:** 1 State Key Laboratory of Quality Research in Chinese Medicine/Macau Institute for Applied Research in Medicine and Health, Macao University of Science and Technology, Macao, China; 2 BGI-Shenzhen, Shenzhen 518083, China; 3 China National Genebank, BGI-Shenzhen, Shenzhen 518120, China; 4 International Institute for Translational Research of Traditional Chinese Medicine of Guangzhou University of Chinese Medicine, Guangzhou, Guangdong 510006, China; 5 Fujian University of Traditional Chinese Medicine, No.1, Qiuyang Road, Minhoushangjie, Fuzhou, Fujian 350122, China; 6 BGI Education Center, University of Chinese Academy of Sciences, Shenzhen 518083, China; 7 Shenzhen Engineering Laboratory of Detection and Intervention of human intestinal microbiome, BGI-Shenzhen, Shenzhen 518083, China; 8 Shenzhen Key Laboratory of Human Commensal Microorganisms and Health Research, BGI-Shenzhen, Shenzhen 518083, China; 9 Laboratory of Genomics and Molecular Biomedicine, Department of Biology, University of Copenhagen, 2100 Copenhagen, Denmark

## Abstract

**Background:**

Laboratory rats such as the Sprague-Dawley (SD) rats are an important model for biomedical studies in relation to human physiological or pathogenic processes. Here we report the first catalog of microbial genes in fecal samples from Sprague-Dawley rats.

**Findings:**

The catalog was established using 98 fecal samples from 49 SD rats, divided in 7 experimental groups, and collected at different time points 30 days apart. The established gene catalog comprises 5,130,167 non-redundant genes with an average length of 750 bp, among which 64.6% and 26.7% were annotated to phylum and genus levels, respectively. Functionally, 53.1%, 21.8%,and 31% of the genes could be annotated to KEGG orthologous groups, modules, and pathways, respectively.

**Conclusions:**

A comparison of rat gut metagenome catalogue with human or mouse revealed a higher pairwise overlap between rats and humans (2.47%) than between mice and humans (1.19%) at the gene level. Ninety-seven percent of the functional pathways in the human catalog were present in the rat catalogue, underscoring the potential use of rats for biomedical research.

## Background

The gut microbiota residing in the human colon is a complex ecological community, which is crucial for a multitude of biological processes [1, 2]. Detailed analyses of the gut microbiota using next-generation sequencing technologies have provided a large amount of information on the composition and gene content of the human gut microbiota and led to the identification of changes associated with a number of human diseases [3–5], the identification of gut microbial markers of importance for early non-invasive diagnosis [6], and even prediction of therapeutic outcomes [7, 8]. Even though the fecal microbiota differs from the microbiota in the upper parts of the digestive tract, fecal samples represent an available proxy for the microbiota in other locations of the gut, and the potential in relation to using signatures or markers of the fecal microbiota for diagnosis and stratification of patients clearly warrants further studies including the use of well-characterized animal models and critical evaluations of the possible use of metagenomic analyses of human fecal samples for use in clinics [9]. Studies of host-microbe interactions in humans have limitations in terms of collection of tissue samples and experimental protocols. Thus, comprehensive animal studies are essential for gaining more knowledge of the importance and function of the gut microbiota, for understanding host-microbiota interactions, and for pre-clinical studies [10, 11].

Rheumatoid arthritis (RA) is a devastating immune disorder with poorly defined etiologies and no curative treatments [12]. Cross-sectional studies have revealed perturbations of the oral and gut microbial communities in RA patients that were partly reversed after treatment [5]. Probiotic supplementation also shows an improvement in RA therapy [7], indicating that microbiota has close correlation in the occurrence, progression, and treatment of RA. Animal models such as adjuvant-induced arthritis (AIA), one of the most widely accepted animal models [13–16], may provide new knowledge on the relationship between the microbiota and RA and possibly contribute to the development of novel microbial-based drugs.

The rat (*Rattus norvegicus*) is one of the most widely and frequently used laboratory animals. Germ-free (GF) rats have been used to explore host-microbiota interactions and examine possible roles of the microbiota in relation to metabolic disorders [16], replantation [17], inflammatory responses [18], and immune processes [19]. However, GF rodents are immune compromised, and thus, the use of GF animals in preclinical work does not directly mimic the human condition. Sprague-Dawley (SD) is one of the most widely used outbred rats in biomedical research, known for its genetic variability. It is extensively used to develop animal models of human conditions such as diabetes [17], obesity [18], cancer [19], and cardiovascular diseases [20], and AIA could also be induced in SD rats. To enable more comprehensive studies of the development and the function of the gut microbiota, detailed catalogs of the gut microbial genes are needed.

The gut microbiota profile of SD rats has been found to be more similar to that of humans than the microbiota profile of mice using 16S rRNA gene amplicon sequencing [21]. Here, we collected fecal samples from SD rats to establish a gut microbial gene catalog using BGISEQ-500-based whole-metagenome shotgun sequencing for the first time. As the composition of the microbiota varies markedly with age, diet, and immune environment, we include information on these different factors to provide a useful reference for future studies including research on AIA arthritis using a SD rat animal model.

## Data Description

Forty-nine male SD rats, 4 weeks of age and weighing approximately 60 g, were purchased from Guangdong Medical Laboratory Animal Center (Guangzhou, China). The rats were randomly divided into 7 groups of 7 rats using a random number table. The groups were: a reference group fed a regular (low-fat) chow, reference group of AIA rats, a group of AIA rats receiving *Lactobacillus casei* (2*10^8^ CFU/d), a group of AIA rats receiving methotrexate (MTX, 7.6mg/kg/week), a group of AIA rats receiving GJK (24 g/kg/d), and a group of AIA rats receiving ZQFTN (50mg/kg/d). The latter 5 groups were all fed the regular (low-fat) chow. In addition, a group of AIA rats were fed a high-fat diet (D12492). All groups had access to feed and water ad libitum. The rats were maintained in individually ventilated cages at 25°C with a humidity of 55% and a 12-h-light/-darkcycle. MTX is a widely used disease-modifying, anti-rheumatic drug [22]. GJK is a Chinese experimental herb formula [23], and ZQFTN is a monomer drug derived from the Chinese traditional herb-*Caulis Sinomenii* [24]. These three drugs have been used in China for RA therapy for a long time with good effectiveness. The rats were acclimated for 14 days to adapt to the laboratory environment before AIA. On day 0 of the experience, we collected fecal samples from the all rats, and subsequently AIA treatment was instigated by a single subcutaneous injection of 0.1 mL of complete Freund's adjuvant (CFA) containing 0.2 mg of *Mycobacterium tuberculosis* H37Ra (BD, Sparks, USA) and mineral oils (Sigma-Aldrich, Milwaukee, USA) into the root of rats' tails [15]. An equal volume of saline was injected into the reference groups. From day 0 to day 30, rats were gavaged daily with *L. casei* (2*10^8^ CFU/d), MTX (7.6 mg/kg/week),GJK (24 g/kg/d),or ZQFTN (50mg/kg/d). The regular (low-fat) chow reference group, the AIA (low-fat) chow group, and the AIA high-fat diet group were given 0.3% CMC-Na. Body weights were determined every 3 days (Table S1). On days 7, 14, 21, and 30, we collected fecal samples from all rats, and the rats were sacrificed on day 30 by cervical dislocation. All the collected fecal samples were immediately placed into drikold for preservation.

**Figure 1:**
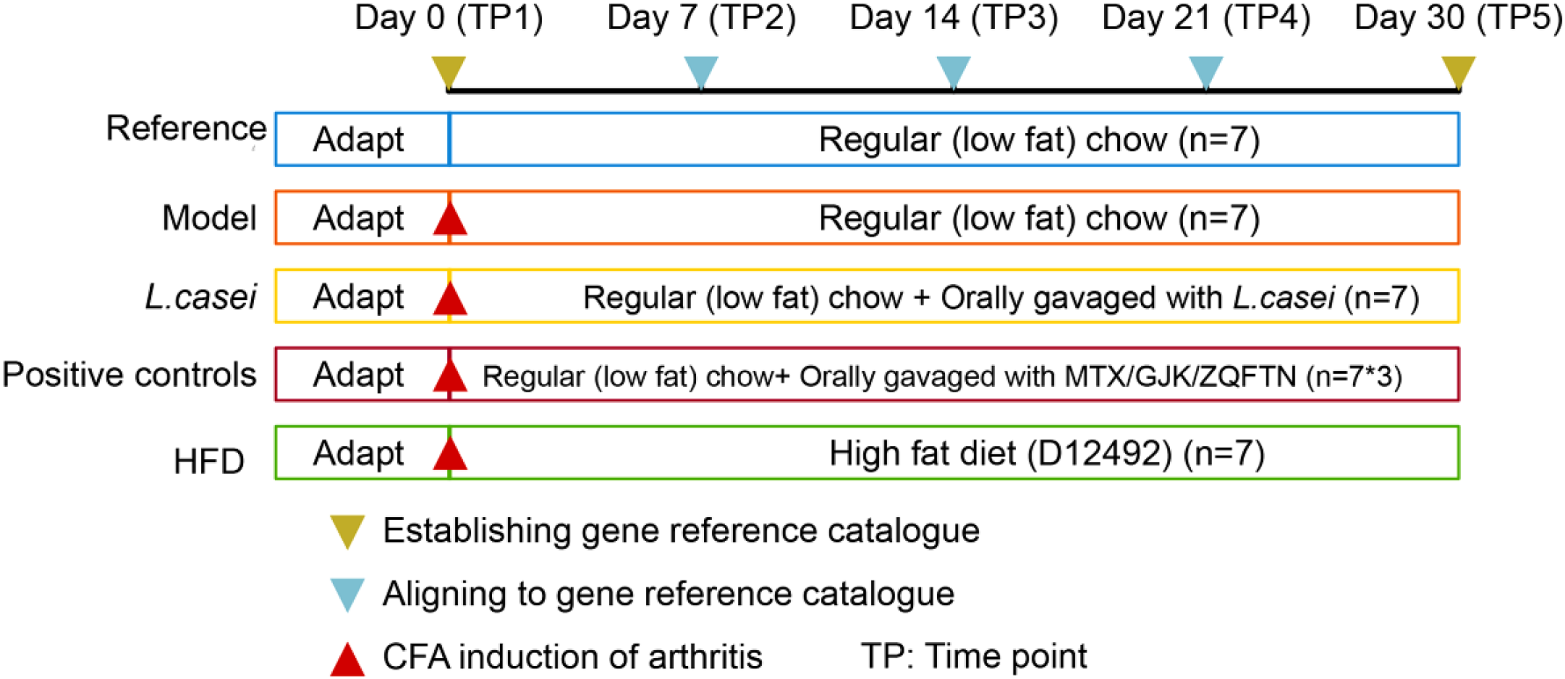
Experimental setup and fecal samples for establishment and assessment of the gene catalog of the gut microbiome in SD rats. Forty-nine SD rats were grouped into seven groups comprising a reference group (n = 7) fed a regular (low-fat) chow, a group of AIA rats treated with vehicle (n = 7), a group of AIA rats gavaged with *L. casei* (n = 7), a group of AIA rats gavaged with MTX (n = 7), a group of AIA rats gavaged with GJK (n = 7) or ZQFNT (n = 7),and a group of AIA rats fed a high-fat diet (D12492) (n = 7). Arthritis was induced by injection of CFA on day 0 after fecal sample collection; fecal samples were collected for five time points (TP) on day 0 (TP1), day 7 (TP2), day 14 (TP3), day 21 (TP4), and day 30 (TP5).

The experimental setup and collection of fecal samples are shown in Fig. 1. We used the 98 fecal samples collected on day 0 and day 30 to establish the reference gene catalog and the remaining 147 samples to assess the quality of the established gene set.

### DNA extraction

Fecal samples were thawed on ice and DNA extraction was performed using the QIAamp DNA Stool Mini Kit (Qiagen, Valencia, CA, USA). Extracts were treated with DNase-free RNase to eliminate RNA contamination. DNA quantity was determined using a Qubit 3.0 fluorometer with the Quant-iT dsDNA BR Assay Kit. The integrity of DNA was evaluated by gel electrophoresis [25].

### Library construction and sequencing

We constructed a sequencing library following the BGISEQ-500 instruction and using the standard protocol with minor modification [26]. In brief, the genomic DNA was fragmented and DNA fragments between 100 bp and ∼300 bp were selected. The selected DNA fragments were repaired and modified. A dTTP tailed adapter sequence was ligated to both ends of the DNA fragments, and the fragments were further amplified and subjected single-strand circularization.

Two types of sequencing strategies, paired-end (PE) and single-end (SE), were followed using the BGISEQ-500 platform with read length of 50 bp and 100 bp, respectively (insert size ∼250bp). In total, we generated 12,621,796,886 reads of PE50 and 11,654,248,439 reads of SE100, representing 2512.6 Gb of raw data (Tables S2 and S3).

### Data preprocessing

High-quality reads will improve performance of metagenomic assembly [27]. To remove or trim low-quality reads, we used our in-house Perl script [28] and the quality was assessed by Phred quality score. The following steps were performed:

- Reads containing more than 3 “N” bases were removed;
- Contiguous bases counted from 3'-end of a read; those with a quality value <20 were trimmed;
- After steps i and ii, the reads with a minimum length of 90 bp and of 40nt forSE reads and PE reads, respectively, were kept.

As expected, a large proportion of BGISEQ-500 generated sequences; 95.93% ∼ 98.80% and 96.47% ∼ 98.61% for SE100 and PE50 reads, respectively, remained as high-quality reads. Further, we aligned clean reads to host genomics DNA (NCBI accession no. NC_005100) by using SOAP aligner v2.22 and an average 9.76% clean reads of SE100 and 11.2% clean reads of PE50 corresponding to host(rat) genome were removed. Thus, we obtained total of high-quality data corresponding to 1689.24 Gb for SE100 and 534.69 Gb for PE50, with an average of 5.21 Gb per sample (Tables S2 and S3) [28, 29].

### Metagenomics sequences de novo assembly

High-quality reads from each DNA samples of day 0 and day 30 were selected for de novo assembly of each sample. We merged high-quality reads of PE50 and SE100 from each sample and assembled them into longer contigs using the IDBA-UD(v1.1.3) by iterated Kmer [30]. Contigs constructed at each round of iteration were used as long-reads for the next iteration with following command line:

idba_ud -r pe.fa -l se.fa –mink 27 –maxk 97 –step 10 -o out_dir –num_threads 24

A total of 67.67% of the reads were assembled into ∼22.9 million contigs with N50 of 5.36 Kb, giving a total contig length of ∼32.3 Gb(Table S2).

### Establishment of a gene catalog of the SD rat gut microbiome

Before performing gene prediction, we filtered the assembled sequences of each of the 98 samples selecting only contigs with a length exceeding 500 bases. These contigs were used for prediction of open reading frames (ORFs) using Prodigal (v2.6.1) with procedure “meta” [31]. In order to bin orthologues and avoid inflation of possible sequencing errors, we grouped shared ORFs using CD-HIT with a criterion of 95% identity >90% of the shorter ORF length with default parameter except “-G 0 –n 8 –aS 0.9 –c 0.95 –d 0 –g 1” [32]. The longest ORF in each group was selected to represent the group, and other members of the group were considered redundant.

**Figure 2:**
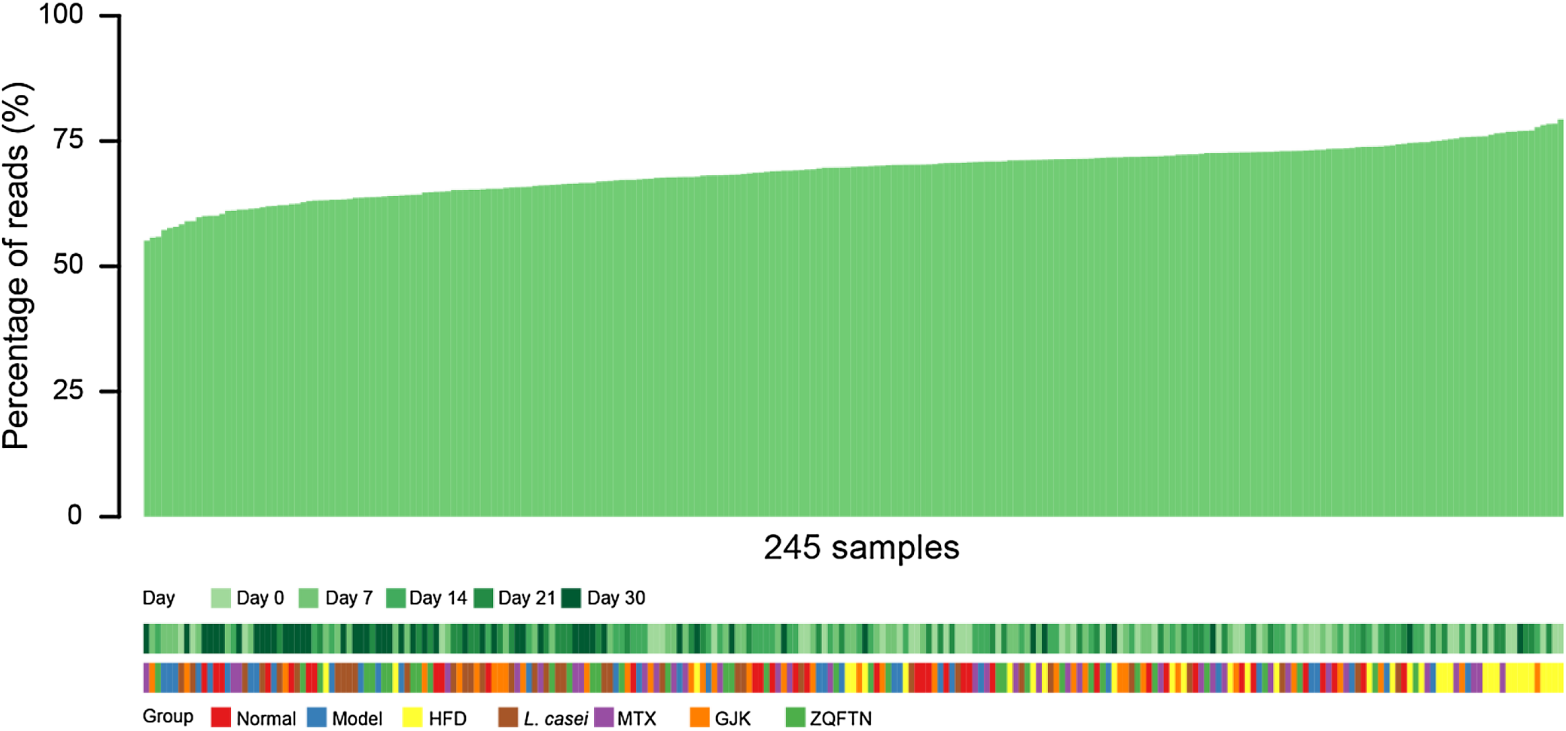
The gene catalog of the gut microbiome in SD rats. Percentage of total reads in this study (n = 245 samples) that could be mapped to gene catalogues of the gut microbiome in rat (green). Collection time and groups of the samples are shown for reference.

ORFs with a length of <100 bp were removed, yielding a non-redundant gene set containing 5,130,167 ORFs with an average length of 750 bp. To assess the representation of the SD rat gut microbiome in the non-redundant gene set, we aligned the ORFs against the SE100 reads from all 245 samples in 7 groups across the 5 collection TPs, using SOAPaligner2 with a 90% identity threshold. A total of 69.5% of reads could be mapped to our gene set, and these reads were employed to compute the relative abundance of each gene in our catalogue (Fig. 2, Table S3).

When accounting for the samples cluster based on gene counts and genus counts in the seven groups, a principal coordinates analysis of the abundance profiles at the level of gene or genera could not clearly separate the gut microbiome in the groups, except for the high-fat diet group (Fig. S1).

### Gene richness

**Figure 3:**
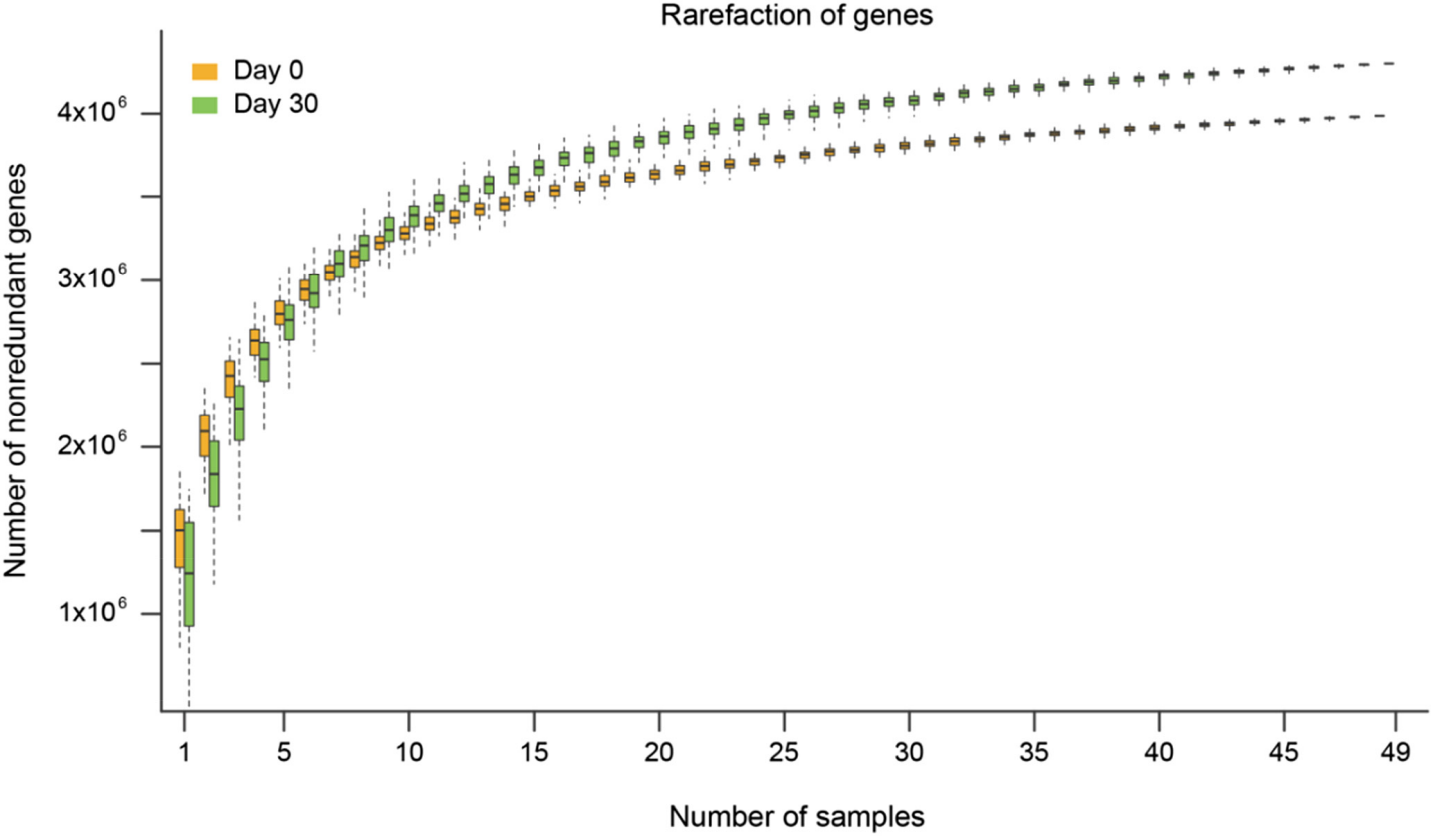
Rarefaction of genes in fecal samples on day 0 and day 30. The number of non-redundant genes was detected along with the increasing numbers of samples (n = 49 for each time point). Yellow: fecal samples from 49 SD rats on day 0; Green: fecal samples from 49 SD rats on day 30.

For a given number of samples at day 0 or day 30, we calculated the total number of identified gene after 100 random samplings with replacement. The rarefaction analysis revealed a curve approaching saturation, suggesting that our gene set included most of gut bacterial genes in the SD rat (Fig. 3). Notably, samples at day 30 had higher gene counts than samples at day 0 (Fig. 3). The Chao 2 index was 92.96%.

### Taxonomic assignment

**Figure 4:**
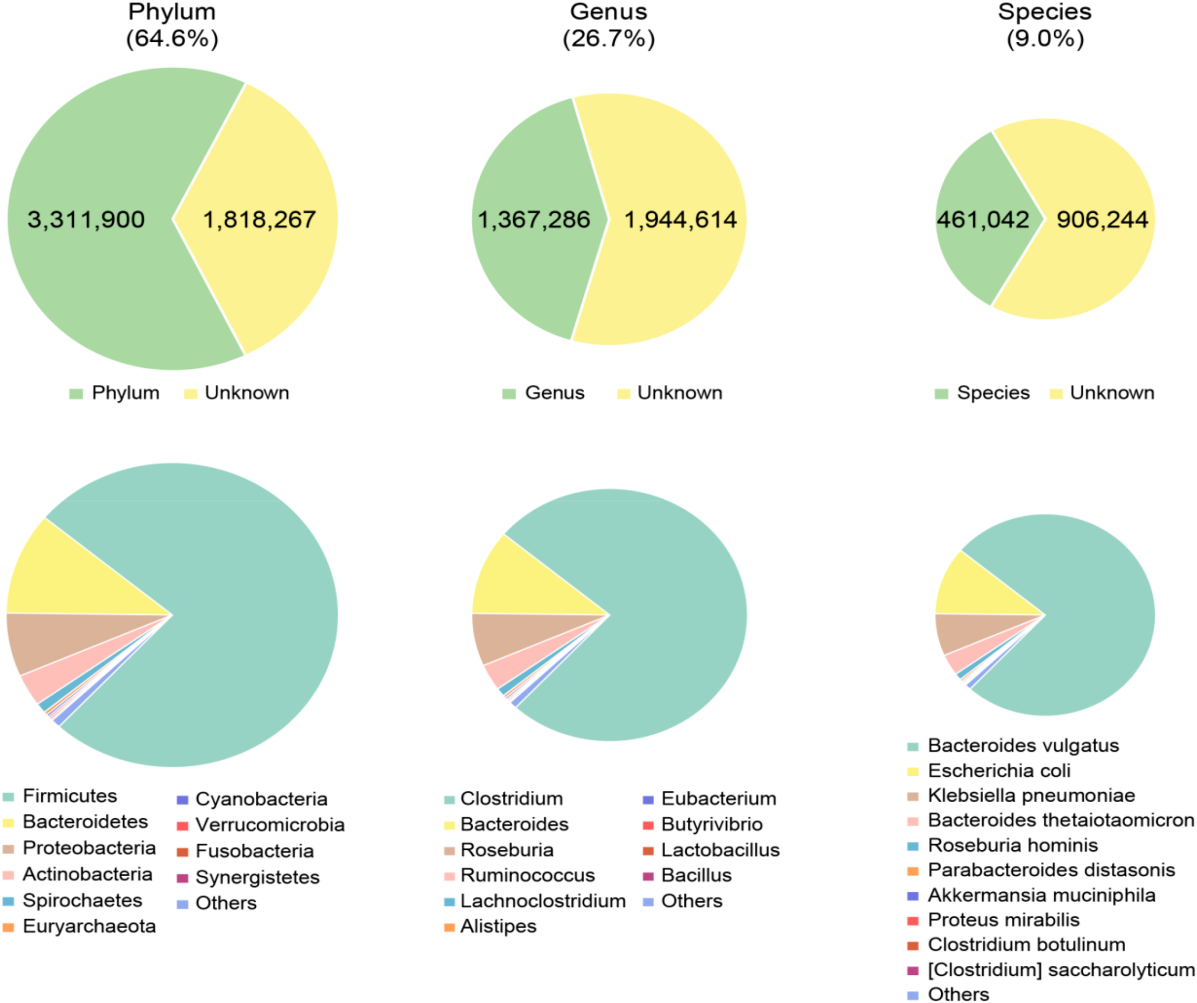
Annotation of the non-redundant genes to phyla, genera, and species. The numbers of non-redundant genes that could be annotated to a phyla, genera, and species are shown with the numbers are shown. The green area reflects the proportion of genes that could be annotated to a phylum, genus, and species. The yellow area reflects unannotated genes. The identity of phyla, genera, and species harboring the annotated genes is displayed below the pie charts.

Taxonomic assignment of the predicted genes was performed using the NCBI-NR database and Integrated Microbial Genomes (v400) database using an in-house pipeline detailed previously [25]. Of the 5,130,167 genes, 64.6% and 26.7% were annotated to the phylum and genus levels, respectively, while only 9% were annotated to the species level (Fig. 4). At the phylum level, most of the annotated genes belonged to Firmicutes (75.90%), followed by Bacteroidetes (10.83%) and Proteobacteria (6.77%)(Fig. 4). At the genus level, the annotated genes (5.30%) primarily belong to *Clostridium* (8.74%), followed by *Bacteroides* (6.25%), *Roseburia* (4.75%), *Ruminococcus* (4.44%),and *Lachnoclostridium* (2.58%), reflecting the paucity of the sequenced rat gut bacterial genomes (Fig. 4).

### Gene functional classification

Putative amino acid sequences were translated from the gene catalogue and searched against the proteins/domains in the KEGG database (release v79.0, with animal and plant genes removed) using BLASTP v2.2.26, with the default parameters except “-m 8 –e 1e-5 –F F –a 6 –b 50.”Each protein was assigned to a KEGG homologue by the highest scoring annotated hit(s) containing at least one high-scoring segment pair scoring over 60 bits.

**Figure 5:**
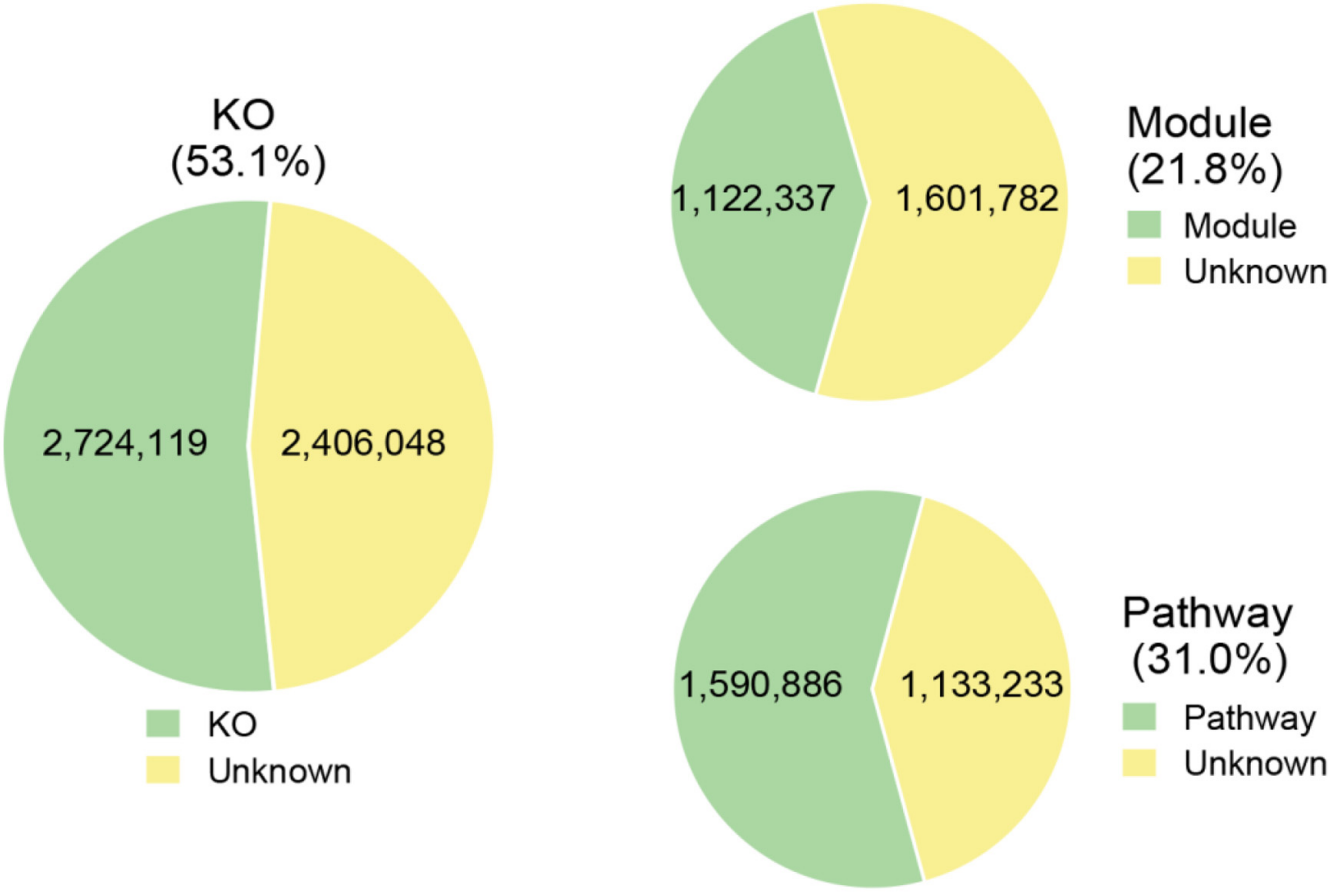
Annotation of non-redundant genes to KOs, modules, and pathways. The numbers of non-redundant genes that could be annotated to KOs, modules, and pathways are shown. The size of the green area reflects the proportion of the genes that could be annotated to KOs, modules, and pathways. The yellow area reflects the proportion of functionally unannotated genes.

Functionally, 53.1%, 21.8%,and 31% of the genes could be annotated to KEGG orthologous groups (KOs), modules, and pathways, respectively (Fig. 5). Among these, we noted metabolic functions, including pathways or modules involved in carbohydrates, amino acid, and energy metabolism; environmental information processing, including membrane transport pathways or modules; and genetic information processing, including replication and repair, translation, and transcription (Tables S4 and S5).

### Comparison of human, mouse, and gene catalogue

**Figure 6:**
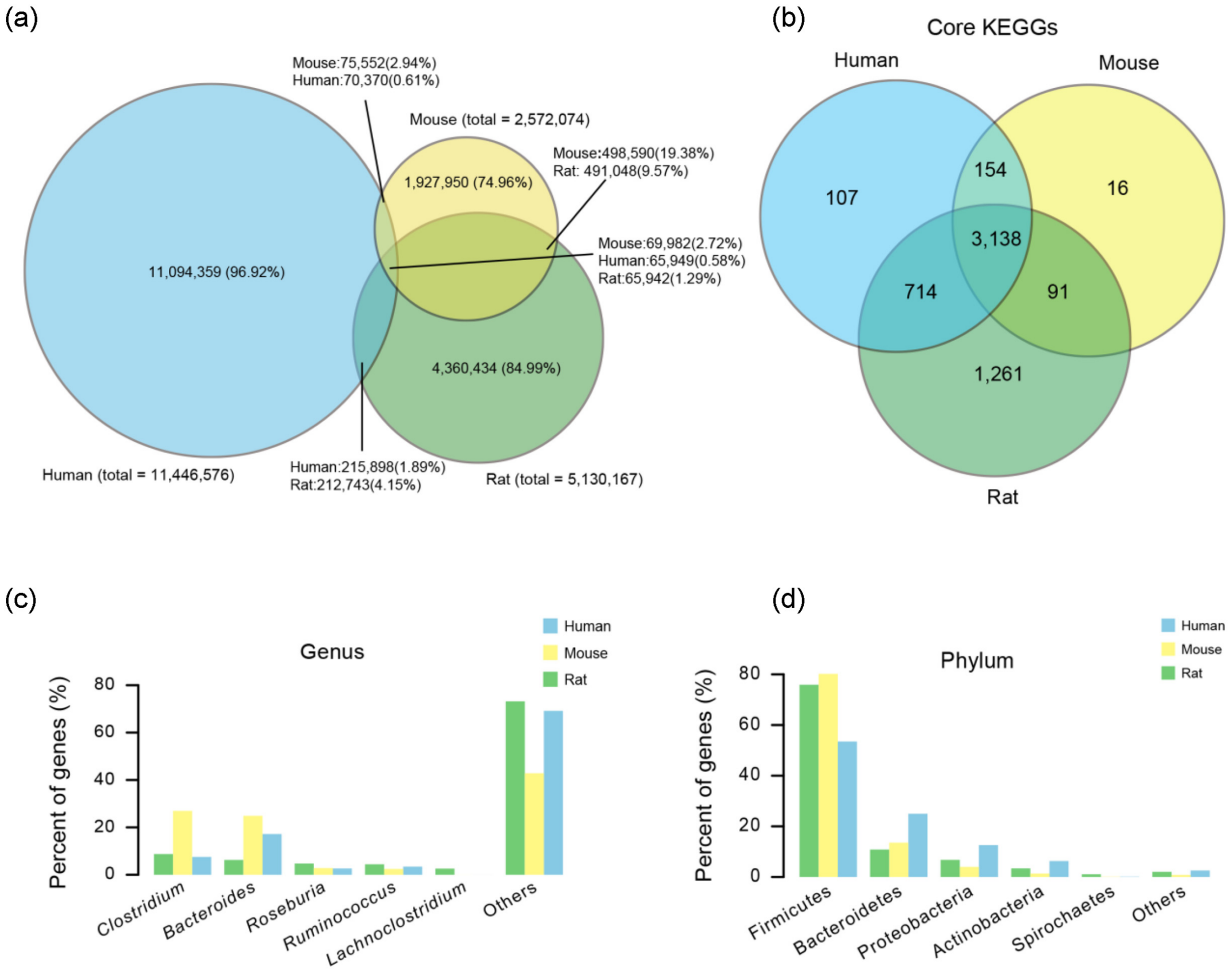
Comparison of the gut microbiome gene catalogs of human, mouse, and rat. **a)** Venn diagram of non-redundant genes shared between human (blue), mouse (yellow), and rat (green) gut microbiome catalogs. **b)** Venn diagram of KO functions shared by the human, mouse, and rat microbiota. **c)** Percentage of genes in genera including *Clostridium, Bacteroides, Roseburia, Ruminococcus*, and*Lachnoclostridium* in the gut microbial gene catalogs of rat, human, and mouse.**d)** The percentages of genes assigned to Fimicutes, Bacteroidetes, Proteobacteria, Aclinobacteria, and Spirochaetes in the gut microbiomes of rat, human, and mouse, respectively.

The rat gut microbial gene catalog was compared to the mouse and the integrated human gut microbial gene catalogs. Only a low percentage of the genes are shared between the rat, human, and mouse catalogs: 1.29% of the genes in the rat gut microbiota, 0.58% of the genes in the human gut microbiota and 2.72% of the genes in the mouse gut microbiota are shared by all three species. The pairwise overlap at the gene level is also modest (rat vs human, 278,685 genes; rat vs mouse 556,990 genes; and mouse vs human 145,534 genes) (Fig. 6a), but was substantially higher for rats and humans (2.47%) than for mice and humans (1.19%). Based on a 90% inter-individual sharing within each animal species, a large proportion of KO functions is shared (3,138 KO identifiers) at the functional level between rat, mouse, and humans (Fig. 6b), representing a functional core in these three mammals. Of note, rats shared more KO identifiers with humans than mice.

To further compare the SD rat gut metagenome catalogue with the mouse and human gut metagenome catalogues, we also aligned all the SE100 reads of the 245 samples to their non-redundant gene set of microbial gene in the human and mouse gut containing ∼11.4 million and ∼2.6 million genes, respectively [33, 34]. An average of 20.45% and 25.41% of the reads of the SD rats mapped to the non-redundant gene sets of the mouse and human gut microbiome, respectively (Table S6). By contrast, as shown in Fig. 2 and Table S3, we observed a much higher mapping ratio of the reads of the 245 samples to non-redundant gene set SD rat, with a mapping average of 69.5, confirming the utility of this reference (Table S6).

We compared the percentage of genes assigned the top six phyla and genera in the three catalogs. Interestingly, the ratios of Firmicutes and Bacteroidetes we observed at the phylum level are similar to those found in mice but markedly different from the human microbiome (Fig. 6c and d).

## Conclusions

The newly established catalogue of the SD rat gut metagenome comprised ∼5.1 million non-redundant genes, which is almost twice the number of microbial gene in the mouse catalog comprising 2.6 million genes established by sequencing samples from different facilities and different mouse strains and also including samples from low-fat-fed as well as high-fat-fed mice. Not surprisingly, the overlap between microbial genes in rat and mouse is larger than between the rodents and humans. However, the overall conclusion based on the available catalogs of genes in the gut microbiota of human [35], mouse [34], rat, and pig [36] points to the remarkable differences in gene sequences in these four mammalian species, implying that a specific catalog for each mammalian species needs to be produced for detailed analyses of the structural and functional analyses of the gut microbiota even though the microbiotas of the four mammals functionally are closely related. Thus, we envisage that the present catalog of genes in the rat gut microbiome will serve as a valuable resource for future work using rats as a model for investigating the role of the gut microbiota and the interactions with the host in health and disease.

### Ethics statement

All experimental procedures were performed in accordance to institutional guidelines for the care and use of laboratory animals in China, and experimental procedures were strictly in accordance with the guidelines for the care and use of laboratory animals (National Research Council of USA, 1996). This study was approved by the Institutional Review Board on Bioethics and Biosafety (Reference number: BGI-FT 16090).

## Availability of supporting data

The sequencing reads from each sequencing library have been deposited at EBI with the accession number: PRJEB22973. The reference catalogue of the 5.1 million genes and related data in this article are available in the *GigaScience* database, GigaDB [37]. All supplementary figures and tables are provided as additional files.

## Additional files

Supplementary Figure 1: A principal coordinates analysis analysis of the 98 samples in 7 groups in gene (a) and genus (b) levels.

Supplementary Table 1: Body weight of the SD rats measured every three days.

Supplemetary Table 2: Metagenomic sequencing data for the SD rat microbiome gene catlogue.

Supplemetary Table 3: Production of single-end reads at day 7, 14, and 21.

Supplemetary Table 4: Reads mapped to human, mouse, or SD rat gut micirobiome gene catalogue are shown.

Supplemetary Table 5: The distribution of the KOs and pathways in the gene catalogue.

Supplemetary Table 6: The distribution of the KOs and modules in the gene catalogue.

## Conflicts of interest

The authors declare no competing financial interests.

## Abbreviations

AIA: adjuvant-induced arthritis; BP: base pairs; CFU: colony-forming unit; GF: germ-free; HSP: high-scoring segment pair; IMG: integrated microbial genomes; KEGG: Kyoto Encyclopedia of Genes and Genomes; KOs: KEGG orthologous groups; L.casei: Lactobacillus casei; MT: Mycobacterium tuberculosis; M: million; MTX: methotrexate; ORFs: open reading frames; PE: paired-end; PCoA: principal coordinates analysis; RA: rheumatoid arthritis; SD: sprague-dawley; SE: single-end

## Funding

This work was financially supported by grants from the Macao Science and Technology Development Fund (Project code: 102/2016/A3), the Shenzhen Municipal Government of China (JSGG20160229172752028, JCYJ20160229172757249 ), and the National Natural Science Foundation of China (Grant No. 81670606).

## Authors' contributions

Design of the study: L. Liu and H.J.; methodology: H.P., R.G., J.Z., Q.W., Y.J., Y.X., Y.Z., Z.W., T.L., Z.L., L. Lu and X.X.; investigation: H.P., Y.Z., R.L., and Z.Y.; data analysis: R.G., J.Z., Q.W., Y.J., F.L. and L.X.; sample collection: H.P., B.T.; writing of the first version of the manuscript: H.P. and R.G.; restructuring and extensive revision of the manuscript: K.K., H.Y., J.W.; supervision of work: L. Liu and H.J.; funding acquisition: L. Liu and H.J.

## Supplementary Material

GIGA-D-17-00275_Original_Submission.pdf

GIGA-D-17-00275_Revision_1.pdf

Response_to_Reviewer_Comments_Original_Submission.pdf

Reviewer_1_Report_(Original_Submission) -- Tue Sparholt Jørgensen 12/5/2017 Reviewed

Reviewer_1_Report_(Revision_1) -- Tue Sparholt Jørgensen 4/2/2018 Reviewed

Reviewer_2_Report_(Original_Submission) -- Raad Gharaibeh 12/7/2017 Reviewed

Reviewer_2_Report_(Revision_1) -- Raad Gharaibeh 3/29/2018 Reviewed

Supplemental material
