# Supplementary material for: A gene catalogue of the Sprague-Dawley rat gut metagenome
Source: Gigascience. 2018 May 11;7(5):giy055. doi: 10.1093/gigascience/giy055 (PMC5967468; doi:10.1093/gigascience/giy055)
Supplement: GIGA-D-17-00275_Original_Submission.pdf [file giy055_giga-d-17-00275_original_submission.pdf]

GigaScience

A gene catalogue of Sprague-Dawley (SD) rat gut metagenome

--Manuscript Draft--

**Manuscript Number:**	GIGA-D-17-00275
**Full Title:**	A gene catalogue of Sprague-Dawley (SD) rat gut metagenome
**Article Type:**	Data Note
**Funding Information:**	Macau Technology Development Fund (102/2016/A3) — Prof. Liang Liu<br>Shenzhen Municipal Government of China (JSGG20160229172752028, JCYJ20160229172757249) — Mrs Huijue Jia
**Abstract:**	Laboratory rats like Sprague-Dawley (SD) are crucial for studying human physiological or pathogenic processes including microbiome. To explore the comprehensively composable and functional alterations on perturbed microbe-host interactions in inflammatory arthritic rats, we establish the gene set of Sprague-Dawley (SD) rat gut metagenome for the first time using ninety-eight fecal samples of 49 SD rats from 7 individual groups at 2 different time points (Day 0 and Day 30). The established gene set comprised 5,130,167 non-redundant genes with an average length of 750 base pairs (BP). 69.5% of reads could be aligned to our gene set, which is higher than the alignment rate for human and mouse gut metagenome. This established gene catalogue of SD rats gut metagenome has adequate reference value in adjuvant-induced arthritis SD rats and can promote further research on the rat gut microbiome.
**Corresponding Author:**	Liang Liu<br><br>CHINA
**Corresponding Author Secondary Information:**	
**Corresponding Author's Institution:**	
**Corresponding Author's Secondary Institution:**	
**First Author:**	Hudan Pan
**First Author Secondary Information:**	
**Order of Authors:**	Hudan Pan
	Ruijin Guo
	Jie Zhu
	Qi Wang
	Yanmei Ju
	Ying Xie
	Yanfang Zheng
	Zhifeng Wang
	Ting Li
	Zhongqiu Liu
	Linlin Lu
	Fei Li
	Chenchen Zhang
	Bin Tong

Powered by Editorial Manager® and ProduXion Manager® from Aries Systems Corporation

	Xiaopng Li
	Yuanqiang Zou
	Liang Xiao
	Xun Xu
	Hui Wang
	Elaine Lai-Han Leung
	Runze Li
	Zhongwen Yuan
	Rutong Ren
	Huanming Yang
	Jian Wang
	Hua Zhou
	Huijue Jia
	Liang Liu
**Order of Authors Secondary Information:**	
**Opposed Reviewers:**	
**Additional Information:**	
**Question**	**Response**
Are you submitting this manuscript to a special series or article collection?	No
**Experimental design and statistics**<br><br>Full details of the experimental design and statistical methods used should be given in the Methods section, as detailed in our Minimum Standards Reporting Checklist. Information essential to interpreting the data presented should be made available in the figure legends.<br><br>Have you included all the information requested in your manuscript?	Yes
**Resources**<br><br>A description of all resources used, including antibodies, cell lines, animals and software tools, with enough information to allow them to be uniquely identified, should be included in the Methods section. Authors are strongly encouraged to cite Research Resource Identifiers (RRIDs) for antibodies, model organisms and tools, where possible.<br><br>Have you included the information requested as detailed in our Minimum	Yes

Powered by Editorial Manager® and ProduXion Manager® from Aries Systems Corporation

Standards Reporting Checklist?	
**Availability of data and materials**<br><br>All datasets and code on which the conclusions of the paper rely must be either included in your submission or deposited in publicly available repositories (where available and ethically appropriate), referencing such data using a unique identifier in the references and in the "Availability of Data and Materials" section of your manuscript.<br><br>Have you have met the above requirement as detailed in our Minimum Standards Reporting Checklist?	Yes

Manuscript

Click here to download Manuscript Rat.geneset.giga.datanote.PHD171013.Ruijin1015.docx

A gene catalogue of Sprague-Dawley (SD) rat gut metagenome

Hudan Pan[1,9], Ruijin Guo[1,2,3,9], Jie Zhu[2,3,9], Qi Wang[2,3,6,9], Yanmei Ju[2,3,9], Ying Xie[1], Yanfang Zheng[1,5], Zhifeng Wang[2,3], Ting Li[1], Zhongqiu Liu[4], Linlin Lu[4], Fei Li[2,3,6], Chenchen Zhang [2,3], Bin Tong[2,3], Xiaoping Li[2,3], Yuanqiang Zou[2,3], Liang Xiao[2,3,7], Xun Xu[2,3], Hui Wang[1], Elaine Lai-Han Leung[1], Runze Li[1], Zhongwen Yuan[1], Rutong Ren[1], Huanming Yang[2,3,8], Jian Wang[2,3,8], Hua Zhou[1], Huijue Jia[2,3,] & Liang Liu[1]

[1]State Key Laboratory of Quality Research in Chinese Medicine/Macau Institute for Applied Research in Medicine and Health, Macao University of Science and Technology, Macao, China

[2]BGI-Shenzhen, Shenzhen 518083, China

[3]China National Genebank, BGI-Shenzhen, Shenzhen 518120, China

[4]International Institute for Translational Research of Traditional Chinese Medicine of Guangzhou University of Chinese Medicine, Guangzhou, Guangdong 510006, China

[5]Fujian University of Traditional Chinese Medicine, No.1, Qiuyang Road, Minhoushangjie, Fuzhou, Fujian 350122, China

[6]BGI Education Center, University of Chinese Academy of Sciences, Shenzhen 518083, China

[7]Shenzhen Engineering Laboratory of Detection and Intervention of human intestinal microbiome, BGI-Shenzhen, Shenzhen 518083, China

[8]James D. Watson Institute of Genome Sciences, Hangzhou 310058, China

[9]These authors contributed equally to this work.

Correspondence and requests for materials should be addressed to Liang Liu (lliu@must.edu.mo) or Huijue Jia (jiahuijue@genomics.cn)

Abstract

Laboratory rats like Sprague-Dawley (SD) are crucial for studying human physiological or pathogenic processes including microbiome. To explore the comprehensively composable and functional alterations on perturbed microbe-host interactions in inflammatory arthritic rats, we establish the gene set of Sprague-Dawley (SD) rat gut metagenome for the first time using ninety-eight fecal samples of 49 SD rats from 7 individual groups at 2 different time points (Day 0 and Day 30). The established gene set comprised 5,130,167 non-redundant genes with an average length of 750 base pairs (BP). 69.5% of reads could be aligned to our gene set, which is higher than the alignment rate for human and mouse gut metagenome. This established gene catalogue of SD rats gut metagenome has adequate reference value in adjuvant-induced arthritis SD rats and can promote further research on the rat gut microbiome.

Background

The microbiota resident in the human colon is a complex ecological community, which has been found to be crucial for biological processes [1, 2]. Enabled by next generation sequencing technologies, we have obtained a large amount of information about the compositions and gene content of the human health or disease related microbial communities [3-5]. However, for ethical or religious considerations, host-microbe interactions are difficult to study in the human and studies have therefore been limited to the analysis of fecal samples without the crosstalk to host physiological or pathogenic processes. Studies in laboratory animal experiments expand our basic knowledge of human life sciences including microbiome and are important to develop a better understanding of gut microbiota in pre-clinical state for human microbiota studies [6, 7].

Rheumatoid arthritis (RA) is a devastating immune disorder with poorly defined etiologies and no curative treatments[8]. Cross-sectional studies have revealed microbial disorder for RA patients which

partly reversed after RA treatment [5], indicating that microbiota has a closely correlation in the occurrence, progression and treatment of RA. Animal models can provide new knowledge on the relationship between microbiome and RA and contribute to innovative microbial drug development, among which Adjuvant-induced arthritis (AA) is one of the most widely accepted animal model [9-12]. Rats (*Rattus norvegicus*) are among the most widely and frequently used laboratory animals. Since 1959 Professor Bengt Erik Gustafsson firstly designed stainless steel isolators to raise germ-free (GF) rats, many researchers used GF rats to explore host-microbiota interactions in life science fields, including metabolic disorder [13], replantation [14], inflammatory response [15] and immune process[16]. Based on humanized gnotobiotic models, inoculation of human gut microbiota into GF rats provide a powerful tool in understanding the causality and effect of gut microbiota in the human-like system. However, as humans are never in a truly GF state, the use of GF animals in preclinical work does not directly mimic the human condition. It is still unknown that how colonies of microbiota are established and changed, how the synergy or inhibition interactions in different microbes shape microbial compositions and how the host affects the populations of microbiota. To study the crosstalk of microbial and host more comprehensively, establishing a comprehensive catalogue of the rat gut metagenome is urgently needed. Sprague-Dawley (SD) is the most widely used outbred rat in biomedical research, known for its genetic variability. It is extensively used to develop animal models of human conditions such as diabetes [17], obesity[18], cancer[19], cardiovascular diseases[20] and adjuvant-induced arthritis could also be induced in SD rats. The gut microbiota profile of SD rats has been found more similar to that of humans than the microbiota profile of mice using 16S gene sequencing[21]. Here, we collected fecal samples from SD rats to build their gut metagenome by BGISEQ-500 whole-metagenome shotgun sequencing for the first time. As metagenome composition

varied markedly with age, diet and immune environment, we provide information on these different factors to give a useful reference for future studies, especially for the research of arthritis on SD rat animal model.

Ethics statement

All experimental procedures were conducted in conformity with institutional guidelines for the care and use of laboratory animals in China, and animal welfare and experimental procedures were strictly in accordance with the guide for the care and use of laboratory animals (National Research Council of USA, 1996). This study has been also approved by the Institutional Review Board on Bioethics and Biosafety (Reference number: BGI-FT 16090).

Data Description

49 Male Sprague-Dawley (SD) rats weighing approximately 60 g (around 4 weeks) were purchased from Guangdong Medical Laboratory Animal Center (Guangzhou, China). The rats were randomly divided into 7 groups using random number table [Normal control, AIA model, *Lactobacillus casei* (*L.casei*) (2*108 CFU/day), MTX (7.6mg/kg/week), GJK (24g/kg/d), ZQFTN (50mg/kg/d) and high fat diet] and maintained in individually ventilated caging(IVC) system under laboratory conditions at 25 °C and with a normal 12 h/12 h light/dark cycle with humidity of 55%. The rats were allowed 14 days to adapt to the laboratory environment before adjuvant arthritis induction. They were fed with standard diet (6/7cages) or high fat diet (high fat diet group, 1/7 cages) and water ad libitum throughout the adaptation and experience process. On day 0 of the experience, we collected fecal samples from the 49 rats for the first time and adjuvant arthritis was induced then in the six groups except normal control group by a single subcutaneous injection of 0.1 mL of complete Freund’s adjuvant (CFA) [containing 0.2mg of Mycobacterium tuberculosis (MT) H37Ra (BD, Sparks, USA), mineral oils [Sigma-Aldrich,

USA] into the root of rat tail[11]. Equal amount of saline was injected to the normal control group. From day 0 to day 30, rats were treated with *L.casei* (2*108 CFU/day), MTX (7.6mg/kg/week), GJK (24g/kg/d) and ZQFTN (50mg/kg/d) respectively. Normal control group, AA model group and high fat diet group were given 0.3% CMC-Na instead. Body weights of the 49 rats were detected every three days (**Table S1**). On day 7, 14, 21 and day 30, we collected fecal samples from the total 49 rats for the other four times and the rats were sacrificed on day 30 by cervical dislocation. All the collected fecal samples were immediately put into drikold to ensure quality.

The source of fecal samples for establishment and assessment the gene catalogue of gut microbiome in SD rats has been shown in **Fig.1**. We used 98 fecal samples on day 0 and day30 to establish the gene reference catalogue and the rest 147 samples to assess the quality of the established gene set.

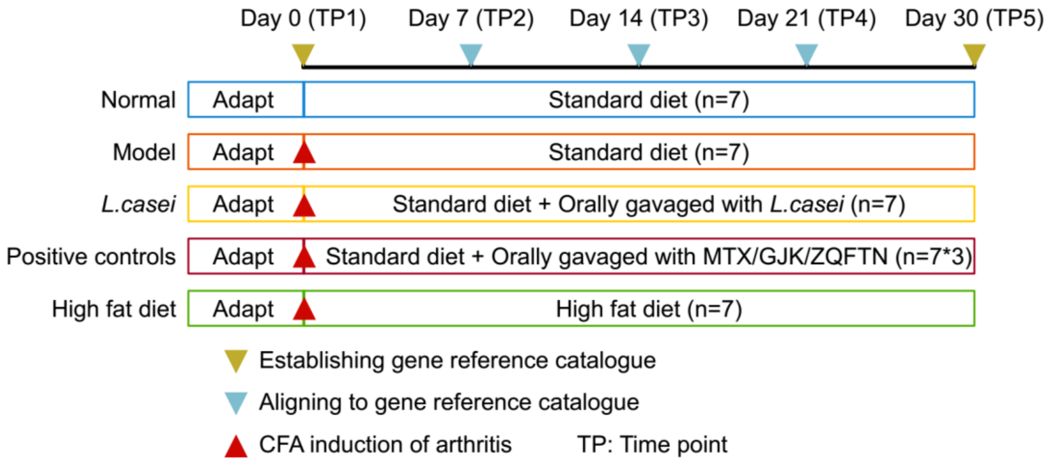

Figure 1. The source of fecal samples for establishment and assessment the gene catalogue of gut microbiome in SD rats. Forty-nine SD rats were grouped into seven groups containing normal rats (n=7), AIA rats treated with vehicle(n=7), *L.casei*(n=7), MTX(n=7), GJK(n=7), ZQFNT(n=7) and AIA(n=7) rats fed with high fat diet. Arthritis was induced by complete Freund’s adjuvant (CFA) on Day 0 after fecal samples collection and fecal samples from the 49 SD rats

were collected for 5 time points (TP) on Day 0 (TP1), Day 7 (TP2), Day 14 (TP3), Day 21 (TP4), Day 30 (TP5).

DNA extraction

Fecal samples were thawed on ice and DNA extraction was performed using the QIAamp® DNA Stool Mini Kit (Qiagen, Valencia, CA, USA). Extracts were treated with DNase-free RNase to eliminate RNA contamination. DNA quantity was determined using Qubit 3.0 fluorometer with the Quant-iT™ dsDNA BR Assay Kit. The integrity of DNA was evaluated on the 2% gel electrophoresis [22].

Library construction and sequencing

We constructed sequencing library followed the BGISEQ-500 instruction. The standard protocol as described in ref, including DNA fragmentation and selection, end repair and a-tailing, and circularization [23].

Two types of sequencing strategies, paired-end (PE) and single-end (SE), was performed on an BGISEQ-500 platform with read length 50 bp (insert size ~250bp) and 100 bp respectively. In total, we generated 12,621,796,886 reads of PE50 and 11,654,248,439 reads of SE100, representing ~2,512.6 Gb of raw data (**Table S2, Table S3**).

Data preprocess

High quality reads will facilitate performance of metagenomic assembly by improve length and accuracy[24]. Briefly, following steps were processed by our in-house Perl script to remove or trim low quality reads[25].

(i) reads that more than 3 of 'N' bases within were removed;

(ii) contiguous bases, which were counted from 3'-end of a read, with quality value less than 3 were trimmed;

(iii) After step i and ii, the reads with minimum length of 90bp and of 40nt for SE reads and PE reads were remained.

As expected, a large proportion of BGISEQ-500 sequence, 95.93% ~ 98.80% and 96.47% ~ 98.61% for reads of SE100 and PE50, respectively, were remained as high-quality reads. Further, we aligned clean reads to host genomics DNA (NCBI accession no. NC_005100) used SOAP aligner v2.22 and an average 9.76% clean reads of SE100 and 11.2% clean reads of PE50 corresponding to host(rat) genome were removed. Finally, we obtained total high-quality data of ~1,689.24Gb for SE100and ~534.69 Gb for PE50, with an average of 5.21 Gb per sample (**Table S2, Table S3**) [25, 26].

Metagenomics sequences de novo assembly

High-quality reads from each DNA samples of Day 0 and Day 30 were selected to perform de novo assembly independently. We merged high-quality reads of PE50 and SE100 from one sample and assembled them into longer contigs using the IDBA-UD(v1.1.3) by iterated Kmer[27]. Contigs constructed at each iteration would be used as long-reads for the next iteration with following command line:

```
idba_ud -r pe.fa -l se.fa --mink 27 --maxk 97 --step 10 -o out_dir --num_threads 24
```

A total of 67.67% of the reads were assembled into ~22.9 million contigs with N50 of 5.36Kb, giving a total contig length of ~32.3Gb (**Table S2**).

Establishment of a catalogue of the SD rat gut metagenome

Before conducting gene prediction, we filtered the assembled sequence of each of the 98 samples and considered only the contigs with a length exceeding 500 bases. These remained contig were therefore subjected to predicted Open reading frames(ORFs) using the Prodigal(v2.6.1) with procedure ‘meta’[28]. In order to fuse orthologues and avoid inflation of possible sequencing errors, we grouped

sharing ORFs using CD-HIT with a criterion of 95% identity over 90% of the shorter ORF length with default parameter except “-G 0 –n 8 –aS 0.9 –c 0.95 –d 0 –g 1”[29]. After this time-consuming process, the longest ORF in each group was selected to represent the group and other members of the group were termed as redundancy.

Finally, the ORFs with length less than 100bp were removed and the non-redundant gene set contained as many as 5,130,167 ORFs with average length of 750 bp. To assess the representation of the SD rat gut microbiome in our non-redundant gene set, we back aligned them by reads of SE100 from all the 245 samples in 7 groups across five collection time-points, using SOAPaligner2 with the 90% identity threshold. A total of 69.5% of reads could be aligned to our gene set and these reads were employed to compute relative abundance of each gene in our catalogue (**Fig. 2, Table S2**).

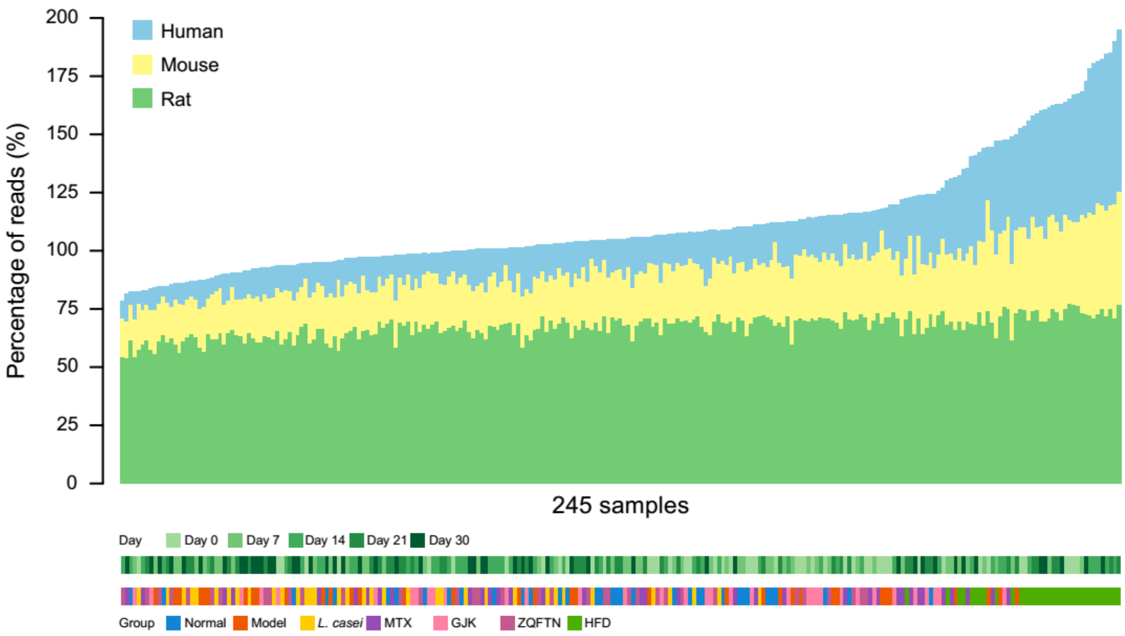

Figure 2. The gene catalogue of the gut microbiome in SD rats Percentage of total reads in this study (n=245 samples) that could be mapped to gene catalogues of the gut microbiome in human (red), mouse (blue) and rat (green). Collection time and groups of the samples are shown for reference.

Gene richness

Rarefaction curve analysis is a method to estimate the richness of gene set, based on the observed gene number in each sampling [22].For a given number of samples at Day0 or Day30, we calculated the total number of identified gene after 100 random samplings with replacement. The rarefaction analysis revealed a curve approaching saturation, suggesting that our gene set includes most of the known SD rat gut bacterial genes (**Fig. 3**). Notably, the samples at Day30 had higher gene count than samples at Day0 (**Fig. 3**).

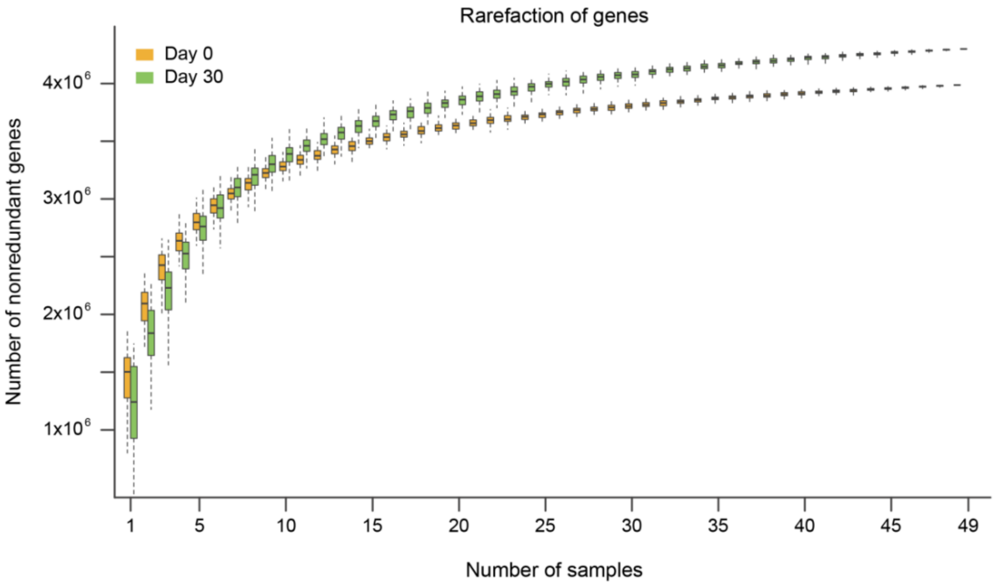

Figure 3. Rarefaction of genes in fecal samples on day 0 and day 30. Amounts of non-redundant genes were detected along with the increasing numbers of samples (n=49 for each time point). Yellow: fecal samples from 49 SD rats on day 0; Green: fecal samples from 49 SD rats on day 30.

Taxonomic assignment

Taxonomic assignment of the predicted genes was done according to the NCBI-NR database and Integrated Microbial Genomes (IMG, v400) database using an in-house pipeline detailed previously[22].Of the 5,130,167 genes, 64.6% and 26.7% were annotated to the phylum and genus levels, respectively, while only 9% were annotated to the species level (**Fig. 4**). At the phylum level,

most of the annotated genes belonged to Firmicutes (75.90%), followed by Bacteroidetes (10.83%) and Proteobacteria (6.77%)(**Fig. 4**). At the genus level, the annotated genes (5.30%) primarily belonged to Clostridium (8.74%), followed by Bacteroides (6.25%), Roseburia (4.75%), Ruminococcus (4.44%) and Lachnoclostridium (2.58%), reflecting the paucity of the sequenced rat gut bacterial genomes (**Fig. 4**).

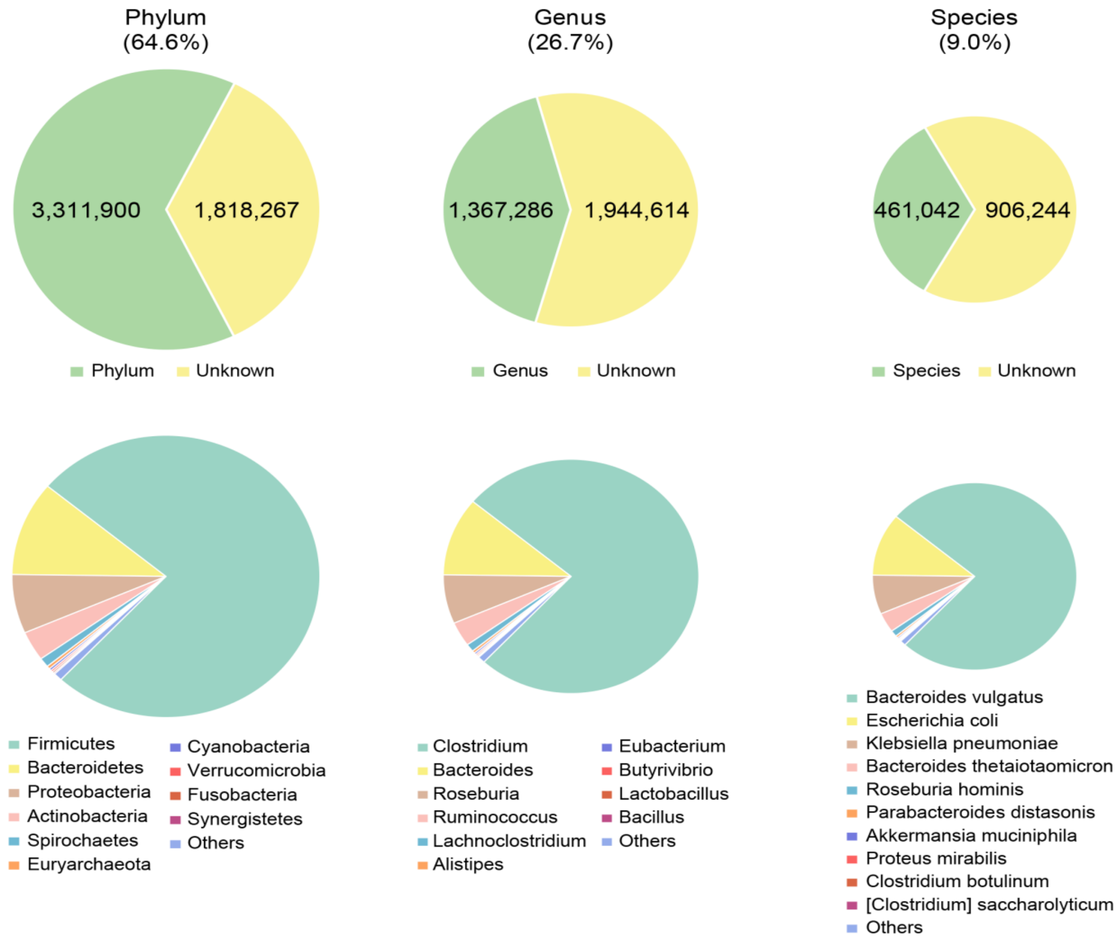

Figure 4. The annotation of the non-redundant genes to phylum, genus and species. The non-redundant genes that could be annotated to phylum, genus and species are shown with the amounts and rates in the upper part. Green: the area of green reflects the rates of the genes that could be annotated to phylum, genus and species. Yellow: the area of yellow reflects unknown genes. The composition of the annotated genes in phylum, genus and species were displayed below, respectively.

Gene functional classification

Putative amino acid sequences were translated from the gene catalogue and searched against the proteins /domains in KEGG database (release v79.0, with animal and plant genes removed) using BLASTP v2.2.26, with the default parameters except "-m 8 –e 1e-5 –F F –a 6 –b 50". Each protein was assigned to a KEGG homologues by the highest scoring annotated hit(s) containing at least on high-scoring segment pair(HSP) scoring over 60 bits.

Functionally, 53.1%, 21.8% and 31% of the genes could be annotated to the KEGG orthologous groups (KOs), modules and pathways, respectively. These results will be beneficial for future rat gut microbiome studies, specifically for SD rats of the same origin (**Fig. 5**).

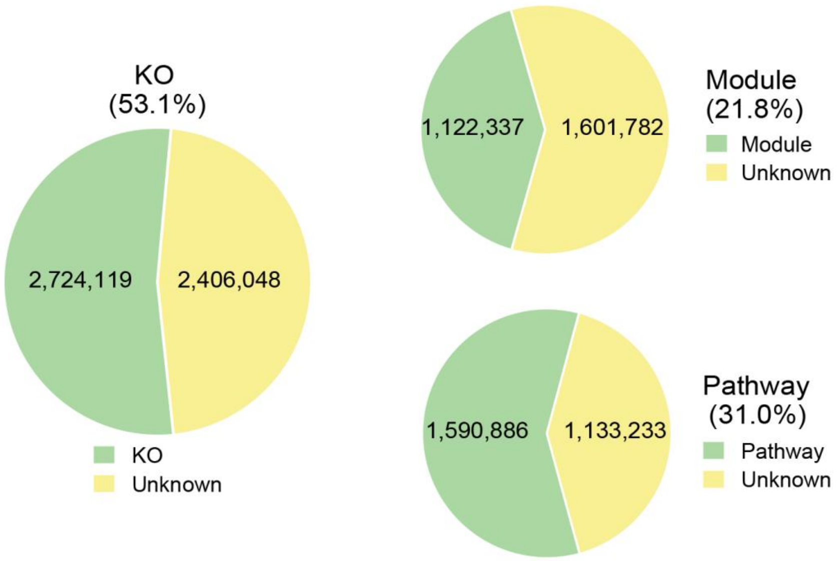

Figure 5. The annotation rates of the non-redundant genes to KO, module and pathway. KO: Kyoto Encyclopedia of Genes and Genomes orthologous groups (KOs). The non-redundant genes that could be annotated to KO, module and pathway are shown with rates. Green: the area of green reflects the rates of the genes that could be annotated to KO, module and pathway. Yellow: the area of white reflects unknown function annotation.

Comparison of human, mouse, rat gene catalogue

To compare the SD rat gut metagenome catalogue with the mouse and the human gut metagenome catalogues, we also aligned all the reads of SE100 of 245 samples to their non-redundant gene set, which contained ~11.4 million and ~ 2.6 million genes, respectively[30, 31]. An average of 20.45% and 25.41% of the reads were allowed for mapping to mouse and human non-redundant gene set (**Fig. 6**). By contrast, a much higher mapping ratio of the reads to SD rat non-redundant gene set, on average 68.82%, confirming the utility of this reference (**Table S4**). Further, we compared the percentage of the genes assigned the top 6 phyla and genera in three catalogues.

Interestingly, the ratios of Firmicutes(75.90%) and Bacteroidetes(10.83%) we observed at the phylum level are similar to those found in mice and are markedly different with the human microbiome (**Fig. 6**).

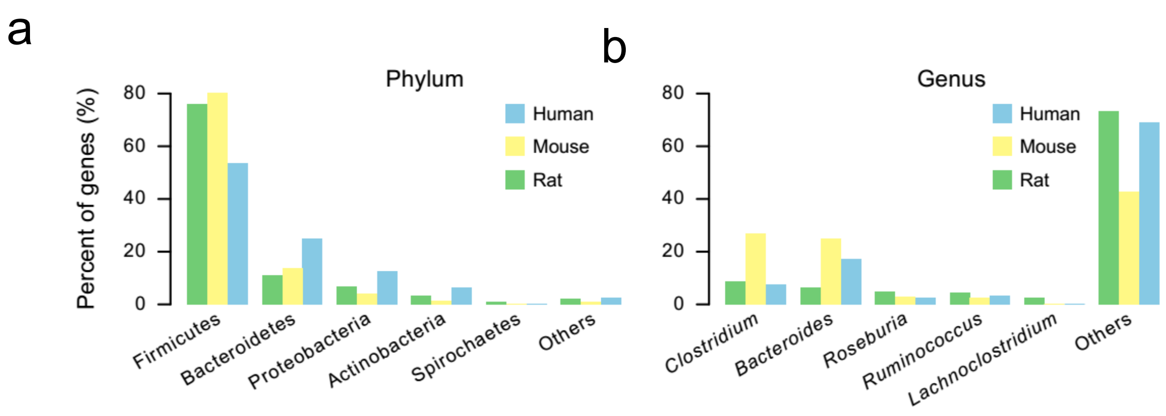

Figure 6. The composition of the rat, human and mouse gut microbiome catalogues at the phylum and genus levels were also compared. (a) In phyla level, percentages of *Fimicutes*, *Bacteroidetes*, *Proteobacteria*, *Aclinobacteria*, *Spirochaetes* and others in rat, human and mouse were also detected in rat, human and mouse respectively. **(b)** Percentage of genes in genera including *Clostridium*, *Bacteroides*, *Roseburia*, *Ruminococcus*, *Lachnoclostridium* and others were compared in the gut microbial gene catalogues of rat, human and mouse.

Conclusions

The newly established catalogue of the SD rat gut metagenome comprised ~5.1 million (M) non-redundant genes, which is more than the published 2.6 M mouse gut microbial gene catalogue. Even with limited rat strains, Sanger sequencing and rearing conditions, the gene catalogue has adequate reference value in AA SD rats and can promote further research on the rat gut microbiome, as its complexity can be enhanced as more samples are analyzed, similar to the human gut metagenome establishing process[30].

Funding

This work was financially supported by grants from the Macau Technology Development Fund (102/2016/A3) and the Shenzhen Municipal Government of China (JSGG20160229172752028, JCYJ20160229172757249).

Availability of supporting data

The sequencing reads from each sequencing library have been deposited at EBI with the accession number: PRJEB22973. The reference catalogue of 5.1 million genes and other related data in this paper are available in the GigaScience database. All supplementary figures and tables are provided in Additional file.

Conflicts of interest

The authors declare no competing financial interests.

Reference

1. Sommer, F., et al., *The resilience of the intestinal microbiota influences health and disease.* Nature reviews. Microbiology, 2017. **15**(10): p. 630-638.

2. Lynch, S.V. and O. Pedersen, *The Human Intestinal Microbiome in Health and Disease.* The New England journal of medicine, 2016. **375**(24): p. 2369-2379.

3. Quince, C., et al., *Shotgun metagenomics, from sampling to analysis.* Nature biotechnology, 2017. **35**(9): p. 833-844.

4. Liu, R., et al., *Gut microbiome and serum metabolome alterations in obesity and after weight-loss intervention.* Nature medicine, 2017. **23**(7): p. 859-868.

5. Zhang, X., et al., *The oral and gut microbiomes are perturbed in rheumatoid arthritis and partly normalized after treatment.* Nature medicine, 2015. **21**(8): p. 895-905.

6. Johnson, C.L. and J. Versalovic, *The human microbiome and its potential importance to pediatrics.* Pediatrics, 2012. **129**(5): p. 950-60.

7. Lloyd-Price, J., G. Abu-Ali, and C. Huttenhower, *The healthy human microbiome.* Genome medicine, 2016. **8**(1): p. 51.

8. McInnes, I.B. and G. Schett, *The pathogenesis of rheumatoid arthritis.* The New England journal of medicine, 2011. **365**(23): p. 2205-19.

9. Waksman, B.H., C.M. Pearson, and J.T. Sharp, *Studies of arthritis and other lesions induced in rats by injection of mycobacterial adjuvant. II. Evidence that the disease is a disseminated immunologic response to exogenous antigen.* Journal of immunology, 1960. **85**: p. 403-17.

10. Pearson, C.M. and F.D. Wood, *Studies of arthritis and other lesions induced in rats by the injection of mycobacterial adjuvant. VII. Pathologic details of the arthritis and spondylitis.* The American journal of pathology, 1963. **42**: p. 73-95.

11. Cai, X., et al., *Manipulation of the induction of adjuvant arthritis in Sprague-Dawley*

rats. Inflammation research : official journal of the European Histamine Research Society ... [et al.], 2006. **55**(9): p. 368-77.

12. Hegen, M., et al., *Utility of animal models for identification of potential therapeutics for rheumatoid arthritis.* Annals of the rheumatic diseases, 2008. **67**(11): p. 1505-15.

13. Arora, T., et al., *Diabetes-associated microbiota in fa/fa rats is modified by Roux-en-Y gastric bypass.* The ISME journal, 2017. **11**(9): p. 2035-2046.

14. Nishioka, M., et al., *Tooth replantation in germ-free and conventional rats.* Endodontics & dental traumatology, 1998. **14**(4): p. 163-73.

15. Anderson, R.C., et al., *Human oral isolate Lactobacillus fermentum AGR1487 induces a pro-inflammatory response in germ-free rat colons.* Scientific reports, 2016. **6**: p. 20318.

16. Patrick, C., et al., *Promotion of autoimmune diabetes by cereal diet in the presence or absence of microbes associated with gut immune activation, regulatory imbalance, and altered cathelicidin antimicrobial Peptide.* Diabetes, 2013. **62**(6): p. 2036-47.

17. Srinivasan, K., et al., *Combination of high-fat diet-fed and low-dose streptozotocin-treated rat: a model for type 2 diabetes and pharmacological screening.* Pharmacological research, 2005. **52**(4): p. 313-20.

18. Levin, B.E., et al., *Selective breeding for diet-induced obesity and resistance in Sprague-Dawley rats.* The American journal of physiology, 1997. **273**(2 Pt 2): p. R725-30.

19. Zhang, R. and L. Lao, *A new rat model of bone cancer pain.* Methods in molecular biology, 2012. **851**: p. 261-73.

20. Delbosc, S., et al., *Involvement of oxidative stress and NADPH oxidase activation in the development of cardiovascular complications in a model of insulin resistance, the fructose-fed rat.* Atherosclerosis, 2005. **179**(1): p. 43-9.

21. Flemer, B., et al., *Fecal microbiota variation across the lifespan of the healthy laboratory rat.* Gut microbes, 2017. **8**(5): p. 428-439.

22. Qin, J., et al., *A metagenome-wide association study of gut microbiota in type 2 diabetes.* Nature, 2012. **490**(7418): p. 55-60.

23. Huang, J., et al., *A reference human genome dataset of the BGISEQ-500 sequencer.* GigaScience, 2017. **6**(5): p. 1-9.

24. Mende, D.R., et al., *Assessment of metagenomic assembly using simulated next generation sequencing data.* PloS one, 2012. **7**(2): p. e31386.

25. Kultima, J.R., et al., *MOCAT: a metagenomics assembly and gene prediction toolkit.* PloS one, 2012. **7**(10): p. e47656.

26. Li, R., et al., *SOAP2: an improved ultrafast tool for short read alignment.* Bioinformatics, 2009. **25**(15): p. 1966-7.

27. Peng, Y., et al., *IDBA-UD: a de novo assembler for single-cell and metagenomic sequencing data with highly uneven depth.* Bioinformatics, 2012. **28**(11): p. 1420-8.

28. Hyatt, D., et al., *Prodigal: prokaryotic gene recognition and translation initiation site identification.* BMC bioinformatics, 2010. **11**: p. 119.

29. Li, W. and A. Godzik, *Cd-hit: a fast program for clustering and comparing large sets of protein or nucleotide sequences.* Bioinformatics, 2006. **22**(13): p. 1658-9.

30. Xie, H., et al., *Shotgun Metagenomics of 250 Adult Twins Reveals Genetic and*

Environmental Impacts on the Gut Microbiome. Cell systems, 2016. **3**(6): p. 572-584 e3.

31. Xiao, L., et al., *A catalog of the mouse gut metagenome.* Nature biotechnology, 2015. **33**(10): p. 1103-8.

Click here to access/download
Supplementary Material
sTable1. The body weight of rats.xlsx

Click here to access/download
Supplementary Material
sTable2.Data for Gene catalogue.xlsx

Click here to access/download
Supplementary Material
sTable3.Reads of SE100 for Alignment.xlsx

Click here to access/download
Supplementary Material
sTable4.Mapping to Catalogues.xlsx
