# Supplementary material for: A gene catalogue of the Sprague-Dawley rat gut metagenome
Source: Gigascience. 2018 May 11;7(5):giy055. doi: 10.1093/gigascience/giy055 (PMC5967468; doi:10.1093/gigascience/giy055)
Supplement: GIGA-D-17-00275_Revision_1.pdf [file giy055_giga-d-17-00275_revision_1.pdf]

GigaScience

A gene catalogue of the Sprague-Dawley rat gut metagenome

--Manuscript Draft--

**Manuscript Number:**	GIGA-D-17-00275R1
**Full Title:**	A gene catalogue of the Sprague-Dawley rat gut metagenome
**Article Type:**	Data Note
**Funding Information:**	Macau Technology Development Fund (102/2016/A3) — Prof. Liang Liu<br>Shenzhen Municipal Government of China (JSGG20160229172752028, JCYJ20160229172757249) — Mrs Huijue Jia<br>National Natural Science Foundation of China (81670606) — Mr Liang Xiao
**Abstract:**	Laboratory rats such as the Sprague-Dawley (SD) rats are important model for biomedical studies in relation to human physiological or pathogenic processes. Here we report on the first catalog of microbial genes in fecal samples from Sprague-Dawley rats. The catalog was established using 98 fecal samples from 49 SD rats divided in 7 experimental groups collected at different time points 30 days apart. The established gene catalog comprises 5,130,167 non-redundant genes with an average length of 750 base pairs (BP), among which 64.6% and 26.7% were annotated to phylum and genus levels, respectively. Functionally, 53.1%, 21.8% and 31% of the genes could be annotated to KEGG orthologous groups (KOs), modules and pathways, respectively. A comparison of rat gut metagenome catalogue with human or mouse revealed a higher pairwise overlap between rats and humans (2.47%) than between mice and humans (1.19%) at the gene level. 97% of the functional pathways in the human catalog were present in the rat catalogue underscoring the potential use of rats for biomedical research.
**Corresponding Author:**	Liang Liu<br><br>CHINA
**Corresponding Author Secondary Information:**	
**Corresponding Author's Institution:**	
**Corresponding Author's Secondary Institution:**	
**First Author:**	Hudan Pan
**First Author Secondary Information:**	
**Order of Authors:**	Hudan Pan
	Ruijin Guo
	Jie Zhu
	Qi Wang
	Yanmei Ju
	Ying Xie
	Yanfang Zheng
	Zhifeng Wang
	Ting Li
	Zhongqiu Liu
	Linlin Lu

Powered by Editorial Manager® and ProduXion Manager® from Aries Systems Corporation

	Fei Li
	Bin Tong
	Liang Xiao
	Xun Xu
	Runze Li
	Zhongwen Yuan
	Huijue Jia
	Liang Liu
	Huanming Yang
	Jian Wang
	Karsten Kristiansen
**Order of Authors Secondary Information:**	
**Response to Reviewers:**	Editorial Office<br>Responses to “GIGA-D-17-00275”<br><br>Thank you for your kind letter of December 8th, 2017. Based on your comment and request, we have made extensive modification on the original manuscript. We hereby send you the revised clean manuscript in the formats of both a PDF file and a MS word file for your consideration. We also include the revised manuscript with all the changes marked in red for easier comparison and editing purposes (see supplemental file).<br>Below follow our responses to the comments and suggestions made by the reviewers.<br>Responses to Reviewer #1:<br>General comment: The data described in this manuscript presents many levels of genetic information from rat feces. It will likely be used as a reference by researchers working in gut function and characterization, and is therefore a valuable contribution to the scientific community. Generally, I find that the manuscript needs English language editing and careful proofreading to weed out small irregularities (e.g. number inconsistencies? on p1.l12) and to make each section more concise (for example, but not only, the section on germ free animals which is not directly related to the present dataset). The experimental and bioinformatical procedure, including sample handling, DNA extraction, assembly, gene prediction, taxonomic assignment, and gene functional annotation is sound, and the descriptions are sufficient.<br>Response: Thanks for your valuable suggestions. We have completely restructured the background section, and hope that it now appears easier to follow and relevant.<br>Specific comments:<br>1. Background, l40: I believe religious considerations are covered by ethics considerations and do not need to be mentioned.<br>Response: We completely agree, and the section has been completely rewritten.<br><br>2. DNA extraction, l26: the sentence "The standard protocol as described in ref, including DNA fragmentation and selection, end repair and a-tailing, and circularization" is not clear.<br>Response: We have revised the section, so it now reads “We constructed sequencing library following the BGISEQ-500 instruction and using the standard protocol with minor modification. In brief, the genomic DNA was fragmented and DNA fragments between 100 base pairs (bp) and ~300 bp were selected. The selected DNA fragments were repaired and modified. A dTTP tailed adapter sequence was ligated to both ends of the DNA fragments and the fragments were further amplified and subjected single-strand circularization.” (line 129-133 in the revised manuscript)<br><br>3. Data preprocess, l56: "quality value less than 3...? which quality measure? Phred-like?<br>Response: We used Phred quality score. The Section has been revised for clarification; it now reads “To remove or trim low quality reads we used our in-house Perl script and the quality was assessed by Phred quality score.” (line 140 in the revised manuscript)

Powered by Editorial Manager® and ProduXion Manager® from Aries Systems Corporation

4. Figure 5; include description of what the modules and pathways consist of somewhere in the text
Response: The compositions of the modules and pathways are now described in the text (line 214-217 in the revised manuscript). The section now reads “Among these, we noted metabolic functions including pathways or modules involved in carbohydrates, amino acid, and energy metabolism; environmental information processing including membrane transport pathways or modules and genetic information processing including replication and repair, translation and transcription (Table S4, S5) .”

5. Comparison of human, mouse, rat gene catalogue, l9: "...%of the reads were allowed for mapping to?. Not understandable
Response: The section has been revised. It now reads “An average of 20.45% and 25.41% of the reads of the SD rats mapped to the non-redundant gene sets of the mouse and human gut microbiome, respectively (Table S6)” (line 238-239 in the revised manuscript).

6. A discussion of the use of fecal samples to evaluate the gut microbiota should be included somewhere.
Response: We added a section discussing this aspect in the Background section (Lines 47-52).
7. A detailed description of the work carried out by each of the 28 authors should also be included, particularly as this is a very high number of authors for a Data Note of limited size and complexity.
Response: We have added a detailed description of the work (line281-287 in the revised manuscript) and removed some authors with fewer contributions. In addition Professor Karsten Kristiansen critically and extensively revised and modified the manuscript, and he is now included a co-author. The final list of authors is shown below:
Hudan Pan1,10, Ruijin Guo1,2,3,10, Jie Zhu2,3,10, Qi Wang2,3,6, Yanmei Ju2,3, Ying Xie1, Yanfang Zheng1,5, Zhifeng Wang2,3, Ting Li1, Zhongqiu Liu4, Linlin Lu4, Fei Li2,3,6, Bin Tong2,3, Liang Xiao2,3,7, Xun Xu2,3Runze Li1, Zhongwen Yuan1,Huanming Yang2,3, Jian Wang2,3 , Karsten Kristiansen2,3,9, Huijue Jia1,2,3,8 & Liang Liu1

Response to Reviewer #2:

General comment: In this manuscript, Pan and colleagues generated a gene set of Sprague-Dawley (SD) rat gut metagenome using 98 stool samples from 49 rats in 7 groups at 2 time-points. The reported set has ~ 5 million non-redundant genes and ~70% of the reads can be aligned to it. The rat gut metagenome catalogue was then compared to that of the mouse and the human gut metagenome catalogues at the phylum and genus levels. This is a useful resource and is of interest to many researchers but I have the following concerns:

Major concerns:
1) Why each sample was assembled alone? No justification is given for this approach. Would assembling all the samples at once produce better assembly (N50, number of genes, etc ..)?
Response: The 98 fecal samples, which produced high-quality reads for assembly, were collected from 49 SD rats at 2 time-points before and after treatment in 7 groups (as shown in Fig 1). A high sequence complexity was expected between pair-wised samples and across all groups. De novo assembly of each sample reduces assembly errors compared to a co-assembling strategy.

2) The authors use the following k-mers 27, 37, 47, 57, 67, 77, 87 and 97 for their assembly. Did the authors run optimizing trials and found those k-mers give the best assembly? Given that their paired-end reads are 50 bases, only three k-mers will be used for PE reads. Would using a lower "--mink" value produce better assembly?
Response: We apologize for this confusion. Actually, two types of reads, 100bp single-end reads and 50bp paired-end reads, were pooled to assemble ilonger contigs using parameter '-r pe.fa -l se.fa'(line134-137 in the revised manuscript). A basic feature of IDBA-UD is the multi k-mer assembly approach which iterates k-mer values in our study from "--mink 27" to "--maxk 97 " by "--step 10" in order to stepwise improve the De-Bruijn graph and the resulting assembly. To balance the number and accuracy of

these assemblies, we adopted this series of k-mer values based on experience that the closest odd number larger or equal to half the average read length should be used.

3) Why pre-correction was not used in IDBA-UD assembly although it is used by IDBA-UD developer for metagenome assembly? Would including "--pre_correction" in IDBA-UD enhances the assembly?
Response: We'd like to thank the Referee for this suggestion. We have now chose reads of 10 samples based on their N50 (5 samples from top rank and 5 samples from bottom rank) and re-assembed them independently by IDBA-UD with parameter " --pre_correction". However, likely due to the sequencing depths our data is not extremely uneven, and we did not observe a significant difference in assembled indices. Please, consult the figure below.
Figure 1 Comparison of "—pre-correction" and "—no pre-correction" in IDBA-UD.

4) A PCA analysis is needed. It is important to know how the samples cluster based on gene counts and taxa counts.
Response: We would like to thank the Referee for this suggestion and we have added a new figure (see below) in the supplementary material as Fig.S1. The text (line174-176), now reads "When accounting for the samples cluster based on gene counts and genus counts in the seven groups, a principal coordinates analysis (PCoA) of the abun¬dance profiles at the level of gene or genera could not clearly separate the gut microbiome in the groups, except for the high fat diet group (Fig. S1)."

Figure 2 A PCoA of the 98 samples of the 7 groups at the gene (a) and the genus (b) levels.

5) Approximate 35% of the genes can't be assigned to any phyla and 47% of the genes can't be assigned to KEGG KOs. How many of those genes overlap (can't be assigned to phyla and can't be assigned to KOs)? Are those misassembled genes?
Response: In our study, we found that approximate 35% of the genes cannot be assigned to any phyla and 47% of the genes cannot be assigned to KEGG KOs. This is comparable to what has been observed for other published gut bacterial gene catalogsIn the rat catalog 1,380,083 genes cannot be assigned to either a KO or a phylum (shown in below). According to early studies which include Sanger sequencing, the misassembly rate was only 0.014 per kb (SOAPdenovo; Qin et al. 2010, Nature.). And by removing redundancy of genes from difference samples (CD-HIT, 95% identity, 90% overlap) com, the potential contribution from misassembly is further reduced. The relative high proportion of genes that cannot be assigned to any phyla or KEGG KOs probably relates to the incomplete coverage of gut microbial genes in the current reference genomes.

Figure 3 Genes that cannot be assigned to a phylum or a KO.
6) A comparison between the rat, mouse and human metagenome genes at the functional level using KEGG KOs, pathways and modules is needed.
Response: We have now included a comparison between the rat, mouse and human metagenome genes at the functional level using KEGG KOs as shown below and in the text Fig.6b.

Figure 4 Venn diagram of score KEGGs shared between the human (blue), mouse (yellow) and rat (green) gut microbiome catalogs.

7) What percentage of genes (not reads) overlap between rat, mouse and human?
Response: We have conducted an analysis determining the of overlap genes (not reads) between rat, mouse and human as shown below and in the text Fig.6a.

Figure 5 Venn diagram of genes shared by the human, mouse and rat catalogs.

8) The reported comparison with human metagenome gene catalogue uses a recent twin study. A comparison using the Human Microbiome project (HMP) gene catalogue is needed since the HMP is the golden standard in the field.
Response: The gene set identified in TwinsUK cohort were merged with the Integrated reference gene catalogue (ICG), leading to an updated gene catalogue containing 11.4 million genes from 1,517 fecal samples of 1,320 people around the world. We believe it is a more comprehensive resource for metagenomics studies on humans. So in this

Powered by Editorial Manager® and ProduXion Manager® from Aries Systems Corporation

	study, we used the human metagenome gene catalogue in as reported in the TwinsUK cohort rather than the Human Microbiome project (HMP) gene catalog.<br><br>Minor concerns:<br>1) Figure 2: figure colors don't match the description given in figure legend. Human in the legend is red but shown as light blue in the figure.<br>Response: We have revised the description in figure legend (line 179 in the revised manuscript). Thanks for the comment.<br><br>2) Figure 5 legend, change white to yellow in: "Yellow: the area of white reflects unknown function annotation"<br>Response: We have revised the figure legend (line 205 in the revised manuscript).<br><br>3) Include a table linking EBI sample IDs to sample IDs shown in supplemental data.<br>Response: We have added a table linking EBI sample IDs shown in supplemental data. Thanks for your suggestion.<br><br>4) Define MTX, GJK and ZQFTN.<br>Response: We have described MTX, GJK and ZQFTN in the revised manuscript (line 96-99 in the revised manuscript). The section now reads “MTX is a widely used disease-modifying anti-rheumatic drug. GJK is a Chinese experimental herb formula and ZQFTN is a monomer drug derived from the Chinese traditional herb-Caulis Sinomenii. These three drugs have been used in China for RA therapy for a long time with good effectiveness.”<br><br>5) There are many typos in the manuscript that need to be addressed, for example, "that" in: "It is still unknown that how colonies of microbiota are established and changed". A carful round of editing is needed.<br>Response: The manuscript has been extensively modified and revised.<br><br>We hope the above our responses and the revised manuscript address the comments and suggestions from the reviewers and we hope that the revised manuscript now is acceptable for publication in GigaScience. We are looking forward to hearing from you.<br><br>Yours sincerely,<br><br>Liang Liu<br>_______________________<br>Prof. Liang Liu, MD., Ph.D.<br>President and Chair Professor of Macau University of Science and Technology,<br>Director of the State Key Laboratory of Quality Research in Chinese Medicine,<br>Macau University of Science and Technology<br>Taipa, Macau, China.<br>Tel: +853-8897 2238<br>Fax: + 853-2882 3312<br>E-mail Address: lliu@must.edu.mo
**Additional Information:**	
**Question**	**Response**
Are you submitting this manuscript to a special series or article collection?	No
**Experimental design and statistics**<br><br>Full details of the experimental design and statistical methods used should be given in the Methods section, as detailed in our Minimum Standards Reporting Checklist. Information essential to interpreting the data presented should be made available in the figure legends.	Yes

Powered by Editorial Manager® and ProduXion Manager® from Aries Systems Corporation

Have you included all the information requested in your manuscript?	
**Resources**<br><br>A description of all resources used, including antibodies, cell lines, animals and software tools, with enough information to allow them to be uniquely identified, should be included in the Methods section. Authors are strongly encouraged to cite Research Resource Identifiers (RRIDs) for antibodies, model organisms and tools, where possible.<br><br>Have you included the information requested as detailed in our Minimum Standards Reporting Checklist?	Yes
**Availability of data and materials**<br><br>All datasets and code on which the conclusions of the paper rely must be either included in your submission or deposited in publicly available repositories (where available and ethically appropriate), referencing such data using a unique identifier in the references and in the "Availability of Data and Materials" section of your manuscript.<br><br>Have you have met the above requirement as detailed in our Minimum Standards Reporting Checklist?	Yes

Manuscript

Click here to download Manuscript RatGeneCatalogue4_5_18.docx

A gene catalogue of the Sprague-Dawley rat gut metagenome

Hudan Pan[1,10], Ruijin Guo[1,2,3,10], Jie Zhu[2,3,10], Qi Wang[2,3,6], Yanmei Ju[2,3], Ying Xie[1], Yanfang Zheng[1,5], Zhifeng Wang[2,3], Ting Li[1], Zhongqiu Liu[4], Linlin Lu[4], Fei Li[2,3,6], Bin Tong[2,3], Liang Xiao[2,3,7], Xun Xu[2,3]Runze Li[1], Zhongwen Yuan[1],Huanming Yang[2,3], Jian Wang[2,3] , Karsten Kristiansen[2,3,9], Huijue Jia[1,2,3,8] & Liang Liu[1]

[1]State Key Laboratory of Quality Research in Chinese Medicine/Macau Institute for Applied Research in Medicine and Health, Macao University of Science and Technology, Macao, China

[2]BGI-Shenzhen, Shenzhen 518083, China

[3]China National Genebank, BGI-Shenzhen, Shenzhen 518120, China

[4]International Institute for Translational Research of Traditional Chinese Medicine of Guangzhou University of Chinese Medicine, Guangzhou, Guangdong 510006, China

[5]Fujian University of Traditional Chinese Medicine, No.1, Qiuyang Road, Minhoushangjie, Fuzhou, Fujian 350122, China

[6]BGI Education Center, University of Chinese Academy of Sciences, Shenzhen 518083, China

[7]Shenzhen Engineering Laboratory of Detection and Intervention of human intestinal microbiome, BGI-Shenzhen, Shenzhen 518083, China

[8]Shenzhen Key Laboratory of Human Commensal Microorganisms and Health Research, BGI-Shenzhen, Shenzhen 518083, China

[9]Laboratory of Genomics and Molecular Biomedicine, Department of Biology, University of Copenhagen, 2100 Copenhagen, Denmark

[10]These authors contributed equally to this work.

Correspondence and requests for materials should be addressed to Liang Liu (lliu@must.edu.mo) or Huijue Jia (jiahuijue@genomics.cn)

Abstract

Background: Laboratory rats such as the Sprague-Dawley (SD) rats are an important model for biomedical studies in relation to human physiological or pathogenic processes. Here we report the first catalog of microbial genes in fecal samples from Sprague-Dawley rats.

Findings: The catalog was established using 98 fecal samples from 49 SD rats, divided in 7 experimental groups, and collected at different time points 30 days apart. The established gene catalog comprises 5,130,167 non-redundant genes with an average length of 750 base pairs (BP), among which 64.6% and 26.7% were annotated to phylum and genus levels, respectively. Functionally, 53.1%, 21.8% and 31% of the genes could be annotated to KEGG orthologous groups (KOs), modules and pathways, respectively.

Conclusions: A comparison of rat gut metagenome catalogue with human or mouse revealed a higher pairwise overlap between rats and humans (2.47%) than between mice and humans (1.19%) at the gene level. 97% of the functional pathways in the human catalog were present in the rat catalogue underscoring the potential use of rats for biomedical research.

Background

The gut microbiota residing in the human colon is a complex ecological community, which is crucial for a multitude of biological processes [1, 2]. Detailed analyses of the gut microbiota using next generation sequencing technologies have provided a large amount of information on the composition and gene content of the human gut microbiota and led to the identification of changes associated with a number of human diseases [3-5], the identification of gut microbial markers of importance for early non-invasive diagnosis [6], and even prediction of therapeutic outcomes [7, 8] Even though the fecal microbiota differs from the microbiota in the upper parts of the digestive tract, fecal samples represent

an available proxy for the microbiota in other locations of the gut, and the potential in relation to using signatures or markers of the fecal microbiota for diagnosis and stratification of patients clearly warrants further studies including the use of well characterized animal models as well as critical evaluations of the possible use of metagenomic analyses of human fecal samples for use in the clinics[9]. Studies of host-microbe interactions in humans have limitations in terms of collection of tissue samples and experimental protocols. Thus, comprehensive animal studies are essential for gaining more knowledge of the importance and function of the gut microbiota, for understanding host-microbiota interactions, and for pre-clinical studies [10, 11].

Rheumatoid arthritis (RA) is a devastating immune disorder with poorly defined etiologies and no curative treatments[12]. Cross-sectional studies have revealed perturbations of the oral and the gut microbial communities in RA patients which were partly reversed after treatment [5] and probiotic supplementation also shows an improvement in RA therapy[7], indicating that microbiota has closely correlation in the occurrence, progression and treatment of RA. Animal models such as adjuvant-induced arthritis (AIA), one of the most widely accepted animal models [13-16], may provide new knowledge on the relationship between the microbiota and RA and possibly contribute to the development of novel microbial-based drugs.

The rat (*Rattus norvegicus*) is one of the most widely and frequently used laboratory animal. Germ-free (GF) rats have been used to explore host-microbiota interactions and examine possible roles of the microbiota in relation to metabolic disorders [16], replantation [17], inflammatory responses [18] and immune processes [19]. However, GF rodents are immune compromised, and thus, the use of GF animals in preclinical work does not directly mimic the human condition. Sprague-Dawley (SD) is one of the most widely used outbred rats in biomedical research, known for its genetic variability. It is

extensively used to develop animal models of human conditions such as diabetes [17], obesity[18], cancer[19], cardiovascular diseases[20] and adjuvant-induced arthritis could also be induced in SD rats. To enable more comprehensive studies of the development and the function of the gut microbiota, detailed catalogs of the gut microbial genes are needed.

The gut microbiota profile of SD rats has been found to be more similar to that of humans than the microbiota profile of mice using 16S rRNA gene amplicon sequencing[21]. Here, we collected fecal samples from SD rats to establish a gut microbial gene catalog using BGISEQ-500-based whole-metagenome shotgun sequencing for the first time. As the composition of the microbiota varies markedly with age, diet and immune environment, we include information on these different factors to provide a useful reference for future studies including research on AIA arthritis using SD rat animal model.

Ethics statement

All experimental procedures were performed in accordance to institutional guidelines for the care and use of laboratory animals in China, and experimental procedures were strictly in accordance with the guidelines for the care and use of laboratory animals (National Research Council of USA, 1996). This study was approved by the Institutional Review Board on Bioethics and Biosafety (Reference number: BGI-FT 16090).

Data description

Forty-nine male SD rats, 4 weeks of age, and weighing approximately 60 g were purchased from Guangdong Medical Laboratory Animal Center (Guangzhou, China). The rats were randomly divided into 7 groups of 7 rats using a random number table. The groups were: a reference group fed a regular (low fat) chow, reference group of AIA rats, a group of AIA rat receiving *Lactobacillus casei* (*L.casei*)

(2*108 CFU/day), a group of AIA rat receiving methotrexate (MTX, 7.6mg/kg/week), a group of AIA rat receiving GJK (24g/kg/d), a group of AIA rat receiving ZQFTN (50mg/kg/d). The latter 5 groups were all fed the regular (low fat) chow. In addition, a group of AIA rat fed a high fat diet (D12492). All groups had access to feed and water ad libitum. The rats were maintained in individually ventilated cages (IVC) at 25 °C with a humidity of 55% and a 12 h/12 h light/dark cycle. MTX is a widely used disease-modifying anti-rheumatic drug[22]. GJK is a Chinese experimental herb formula[23] and ZQFTN is a monomer drug derived from the Chinese traditional herb-*Caulis Sinomenii*[24]. These three drugs have been used in China for RA therapy for a long time with good effectiveness. The rats were acclimated for 14 days to adapt to the laboratory environment before AIA. On day 0 of the experience, we collected fecal samples from the all rats and subsequently AIA treatment was instigated by a single subcutaneous injection of 0.1 mL of complete Freund's adjuvant (CFA) containing 0.2mg of *Mycobacterium tuberculosis* (MT) H37Ra (BD, Sparks, USA), and mineral oils [Sigma-Aldrich, USA] into the root of rat tail[15]. An equal volume of saline was injected into the reference groups. From day 0 to day 30, rats were gavaged daily with *L.casei* (2*108 CFU/day), with MTX (7.6mg/kg/week), with GJK (24g/kg/d) or ZQFTN (50mg/kg/d). The regular (low fat) chow reference group, the AIA (low fat) chow group and the AIA high fat diet group were given 0.3% CMC-Na. Body weights were determined every three days (**Table S1**). On day 7, 14, 21 and day 30, we collected fecal samples from all rats and the rats were sacrificed on day 30 by cervical dislocation. All the collected fecal samples were immediately placed into drikold for preservation.

The experimental setup and collection of fecal samples are shown in **Fig.1**. We used the 98 fecal samples collected on day 0 and day30 to establish the reference gene catalog and the remaining 147 samples to assess the quality of the established gene set.

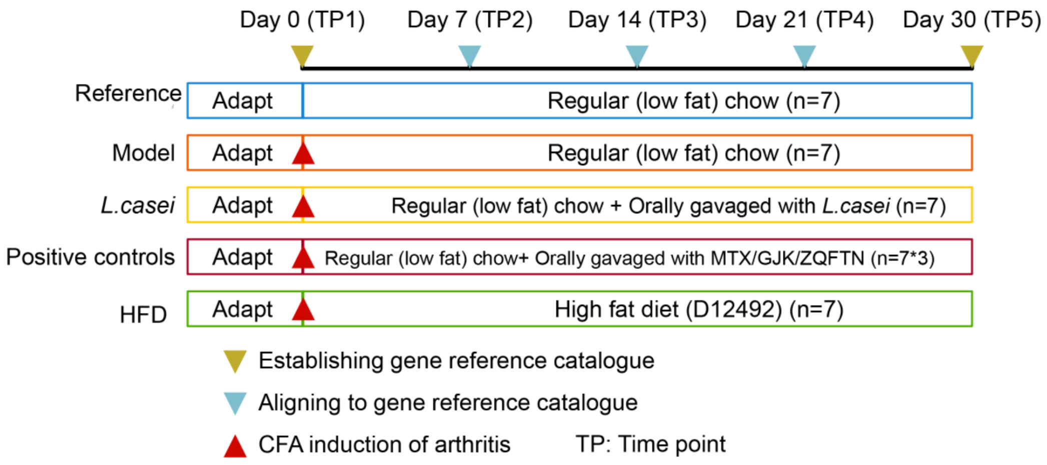

Figure 1. Experimental setup and fecal samples for establishment and assessment the gene catalog of the gut microbiome in SD rats. Forty-nine SD rats were grouped into seven groups comprising a reference group (n=7) fed a regular (low fat) chow, a group of AIA rats treated with vehicle (n=7), a group of AIA rats gavaged with *L.casei* (n=7), a group of AIA rats gavaged *with* MTX (n=7), a group of AIA rats gavaged with GJK (n=7) or ZQFNT (n=7) and a group of AIA rats fed a high fat diet group (D12492)(n=7). Arthritis was induced by injection of complete Freund' s adjuvant (CFA) on Day 0 after fecal samples collection and fecal samples were collected for 5 time points (TP) on Day 0 (TP1), Day 7 (TP2), Day 14 (TP3), Day 21 (TP4) and Day 30 (TP5).

DNA extraction

Fecal samples were thawn on ice and DNA extraction was performed using the QIAamp® DNA Stool Mini Kit (Qiagen, Valencia, CA, USA). Extracts were treated with DNase-free RNase to eliminate RNA contamination. DNA quantity was determined using a Qubit 3.0 fluorometer with the Quant-iT™ dsDNA BR Assay Kit. The integrity of DNA was evaluated by gel electrophoresis [25].

Library construction and sequencing

We constructed sequencing library following the BGISEQ-500 instruction and using the standard protocol with minor modification [26]. In brief, the genomic DNA was fragmented and DNA fragments

between 100 base pairs (bp) and ~300 bp were selected. The selected DNA fragments were repaired and modified. A dTTP tailed adapter sequence was ligated to both ends of the DNA fragments and the fragments were further amplified and subjected single-strand circularization.

Two types of sequencing strategies, paired-end (PE) and single-end (SE), were followed using the BGISEQ-500 platform with read length of 50 bp and 100 bp respectively (insert size ~250bp). In total, we generated 12,621,796,886 reads of PE50 and 11,654,248,439 reads of SE100, representing 2,512.6 Gb of raw data (**Table S2, Table S3**).

Data preprocessing

High quality reads will improve performance of metagenomic assembly [27]. To remove or trim low quality reads we used our in-house Perl script [28] and the quality was assessed by Phred quality score. The following steps were performed:

(i) Reads containing more than 3 'N' bases were removed;

(ii) Contiguous bases counted from 3'-end of a read, with quality value lower than 20 were trimmed;

(iii) After step i and ii, the reads with a minimum length of 90bp and of 40nt for SE reads and PE reads, respectively, were kept.

As expected, a large proportion of BGISEQ-500 generated sequences, 95.93% ~ 98.80% and 96.47% ~ 98.61% for SE100 and PE50 reads, respectively, remained as high-quality reads. Further, we aligned clean reads to host genomics DNA (NCBI accession no. NC_005100) used SOAP aligner v2.22 and an average 9.76% clean reads of SE100 and 11.2% clean reads of PE50 corresponding to host(rat) genome were removed. Thus, we obtained a total of high-quality data corresponding to 1,689.24Gb for SE100and 534.69 Gb for PE50, with an average of 5.21 Gb per sample (**Table S2, Table S3**) [28, 29].

Metagenomics sequences de novo assembly

High-quality reads from each DNA samples of Day 0 and Day 30 were selected for de novo assembly of each sample. We merged high-quality reads of PE50 and SE100 from each sample and assembled them into longer contigs using the IDBA-UD(v1.1.3) by iterated Kmer[30]. Contigs constructed at each round of iteration were used as long-reads for the next iteration with following command line:

idba_ud -r pe.fa -l se.fa --mink 27 --maxk 97 --step 10 -o out_dir --num_threads 24

A total of 67.67% of the reads were assembled into ~22.9 million contigs with N50 of 5.36Kb, giving a total contig length of ~32.3Gb (**Table S2**).

Establishment of a gene catalog of the SD rat gut microbiome

Before performing gene prediction, we filtered the assembled sequences of each of the 98 samples selecting only contigs with a length exceeding 500 bases. These contigs were used for prediction of open reading frames (ORFs) using the Prodigal (v2.6.1) with procedure ‘meta’[31]. In order to bin orthologues and avoid inflation of possible sequencing errors, we grouped shared ORFs using CD-HIT with a criterion of 95% identity over 90% of the shorter ORF length with default parameter except “-G 0 –n 8 –aS 0.9 –c 0.95 –d 0 –g 1”[32]. The longest ORF in each group was selected to represent the group and other members of the group were considered redundant.

ORFs with a length of less than 100bp were removed yielding a non-redundant gene set containing 5,130,167 ORFs with an average length of 750 bp. To assess the representation of the SD rat gut microbiome in the non-redundant gene set, we aligned the ORFs against the SE100 reads from all the 245 samples in 7 groups across the five collection time-points, using SOAPaligner2 with a 90% identity threshold. A total of 69.5% of reads could be mapped to our gene set and these reads were employed to compute the relative abundance of each gene in our catalogue (**Fig. 2, Table S3**).

When accounting for the samples cluster based on gene counts and genus counts in the seven groups, a

principal coordinates analysis (PCoA) of the abundance profiles at the level of gene or genera could not clearly separate the gut microbiome in the groups, except for the high fat diet group (**Fig. S1**).

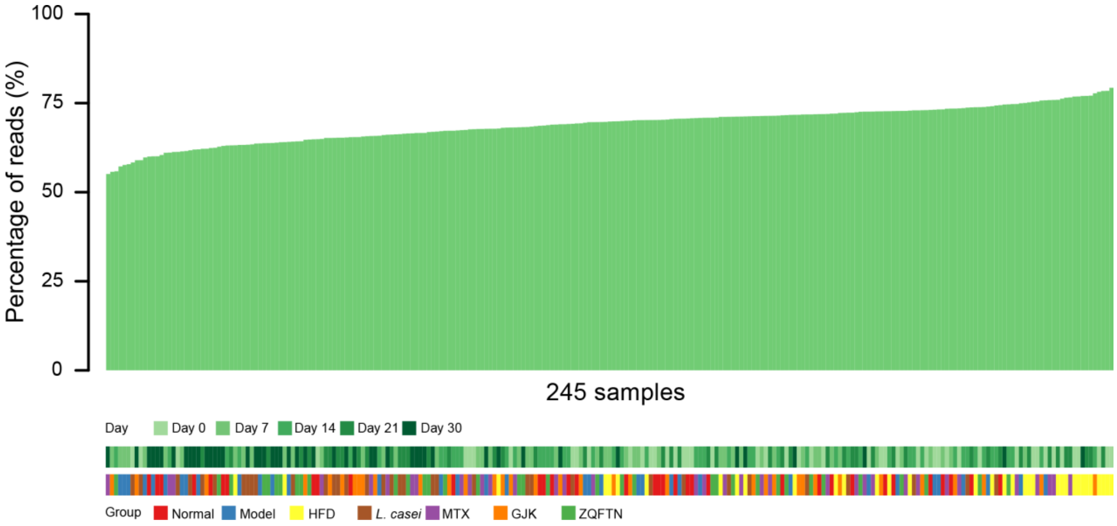

Figure 2. The gene catalog of the gut microbiome in SD rats. Percentage of total reads in this study (n=245 samples) that could be mapped to gene catalogues of the gut microbiome in rat (green). Collection time and groups of the samples are shown for reference.

Gene richness

For a given number of samples at Day0 or Day30, we calculated the total number of identified gene after 100 random samplings with replacement. The rarefaction analysis revealed a curve approaching saturation, suggesting that our gene set included most of gut bacterial genes in the SD rat (**Fig. 3**). Notably, samples at Day30 had higher gene count than samples at Day0 (**Fig. 3**). The Chao 2 index was 92.96%.

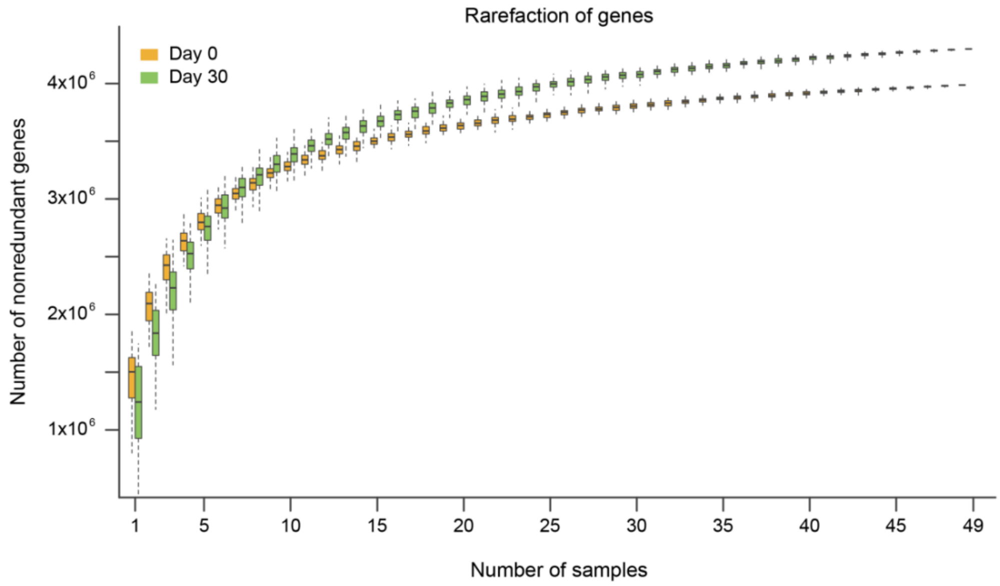

Figure 3. Rarefaction of genes in fecal samples on day 0 and day 30. The number of non-redundant genes were detected along with the increasing numbers of samples (n=49 for each time point). Yellow: fecal samples from 49 SD rats on day 0; Green: fecal samples from 49 SD rats on day 30.

Taxonomic assignment

Taxonomic assignment of the predicted genes performed using the NCBI-NR database and Integrated Microbial Genomes (IMG, v400) database using an in-house pipeline detailed previously[25].Of the 5,130,167 genes, 64.6% and 26.7% were annotated to the phylum and genus levels, respectively, while only 9% were annotated to the species level (**Fig. 4**). At the phylum level, most of the annotated genes belonged to Firmicutes (75.90%), followed by Bacteroidetes (10.83%) and Proteobacteria (6.77%)(**Fig. 4**). At the genus level, the annotated genes (5.30%) primarily belong to *Clostridium* (8.74%), followed by *Bacteroides* (6.25%), *Roseburia* (4.75%), *Ruminococcus* (4.44%) and *Lachnoclostridium* (2.58%), reflecting the paucity of the sequenced rat gut bacterial genomes (**Fig. 4**).

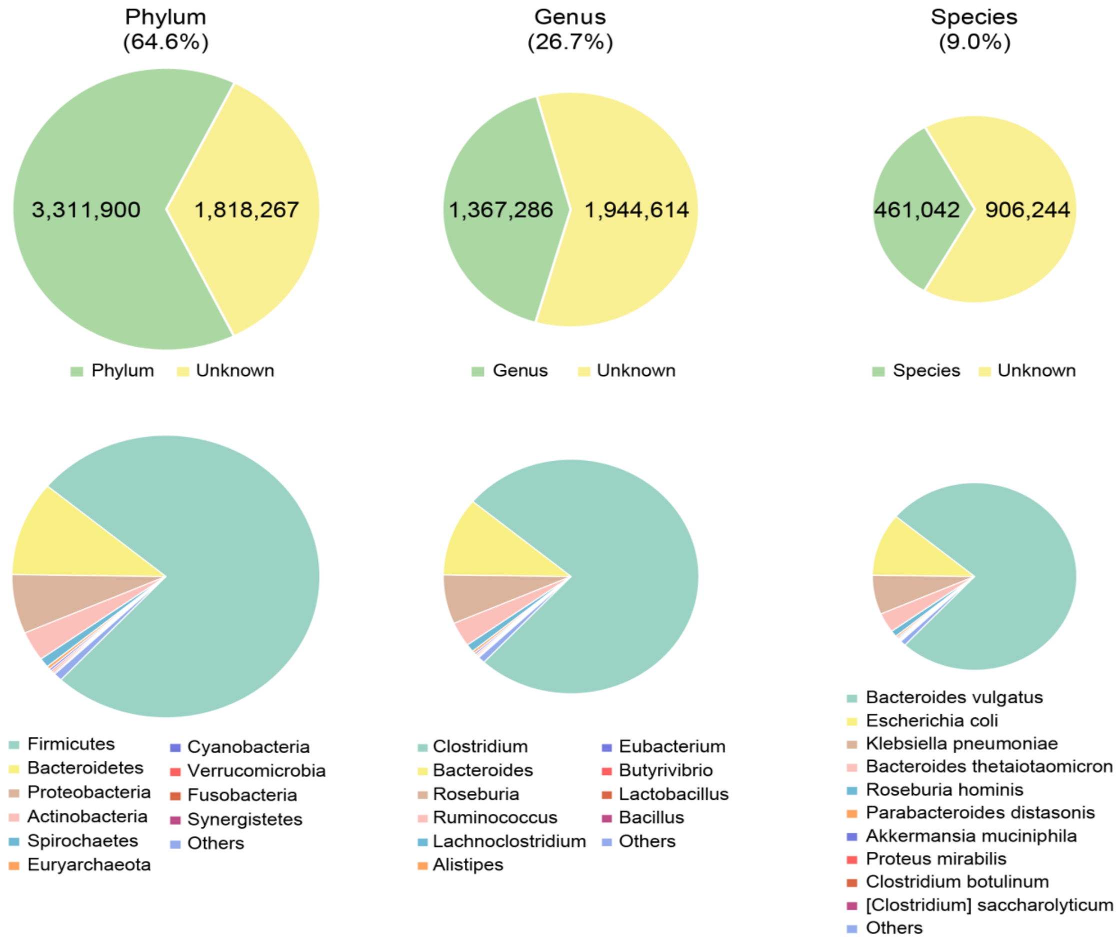

Figure 4. Annotation of the non-redundant genes to phyla, genera and species. The numbers of non-redundant genes that could be annotated to a phyla, genera and species are shown with the numbers are shown. The green area reflects the proportion of genes that could be annotated to a phylum, genus and species. The yellow area reflects unannotated genes. The identity of phyla, genera and species harboring the annotated genes is displayed below the pie charts.

Gene functional classification

Putative amino acid sequences were translated from the gene catalogue and searched against the proteins /domains in the KEGG database (release v79.0, with animal and plant genes removed) using BLASTP v2.2.26, with the default parameters except “-m 8 –e 1e-5 –F F –a 6 –b 50”. Each protein was assigned to a KEGG homologues by the highest scoring annotated hit(s) containing at least on high-scoring segment pair(HSP) scoring over 60 bits.

Functionally, 53.1%, 21.8% and 31% of the genes could be annotated to KEGG orthologous groups (KOs), modules and pathways, respectively (**Fig. 5**). Among these, we noted metabolic functions including pathways or modules involved in carbohydrates, amino acid, and energy metabolism; environmental information processing including membrane transport pathways or modules and genetic information processing including replication and repair, translation and transcription (Table S4,S5) .

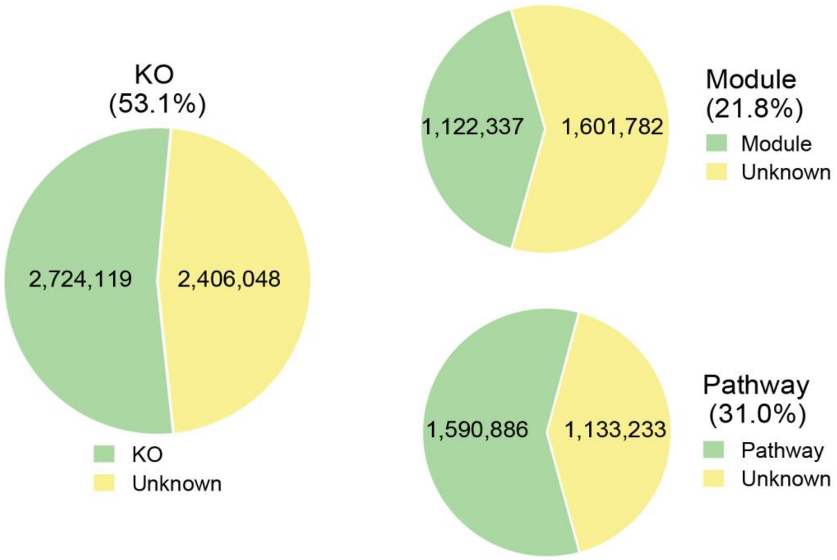

Figure 5. Annotation of non-redundant genes to KOs, modules and pathways. KO: Kyoto Encyclopedia of Genes and Genomes orthologous groups (KOs). The numbers of non-redundant genes that could be annotated to KOs, modules and pathways are shown. The size of green area reflects the proportion of the genes that could be annotated to KOs, modules and pathways. The yellow area reflects the proportion of functionally unannotated genes.

Comparison of human, mouse, rat gene catalogue

The rat gut microbial gene catalog was compared to the mouse and the integrated human gut microbial gene catalogs. Only a low percentages of the genes are shared between the rat, human and mouse catalogs.1.29% of the genes in the rat gut microbiota, 0.58% of the genes in the human gut microbiota and f the genes in the mouse gut microbiota are shared by all three species. The pairwise overlap at the

gene level is also modest (rat versus human, 278,685 genes; rat versus mouse 556,990 genes; and mouse versus human 145,534 genes) (Fig. 6a), but was substantially higher for rats and humans (2.47%) than for mice and humans (1.19%). Based on a 90% inter-individual sharing within each animal species, a large proportion of KEGG orthology (KO) functions is shared (3,138 KO identifiers) at the functional level between rat, mouse and human (Fig. 6b), representing a functional core in these three mammals. Of note rats shared more KO identifiers with human than mice.

To further compare the SD rat gut metagenome catalogue with the mouse and the human gut metagenome catalogues, we also aligned all the SE100 reads of the 245 samples to their non-redundant gene set of microbial gene in the human and the mouse gut containing ~11.4 million and ~ 2.6 million genes, respectively[33, 34]. An average of 20.45% and 25.41% of the reads of the SD rats mapped to the non-redundant gene sets of the mouse and human gut microbiome, respectively (**Table S6**). By contrast, as shown in Fig.2 and Table S3 we observed a much higher mapping ratio of the reads of the 245 samples to non-redundant gene set SD rat, with a mapping average of 69.5, confirming the utility of this reference (**Table S6**).

We compared the percentage of genes assigned the top 6 phyla and genera in the three catalogs. Interestingly, the ratios of Firmicutes and Bacteroidetes we observed at the phylum level are similar to those found in mice, but markedly different from the human microbiome (**Fig. 6c,d**).

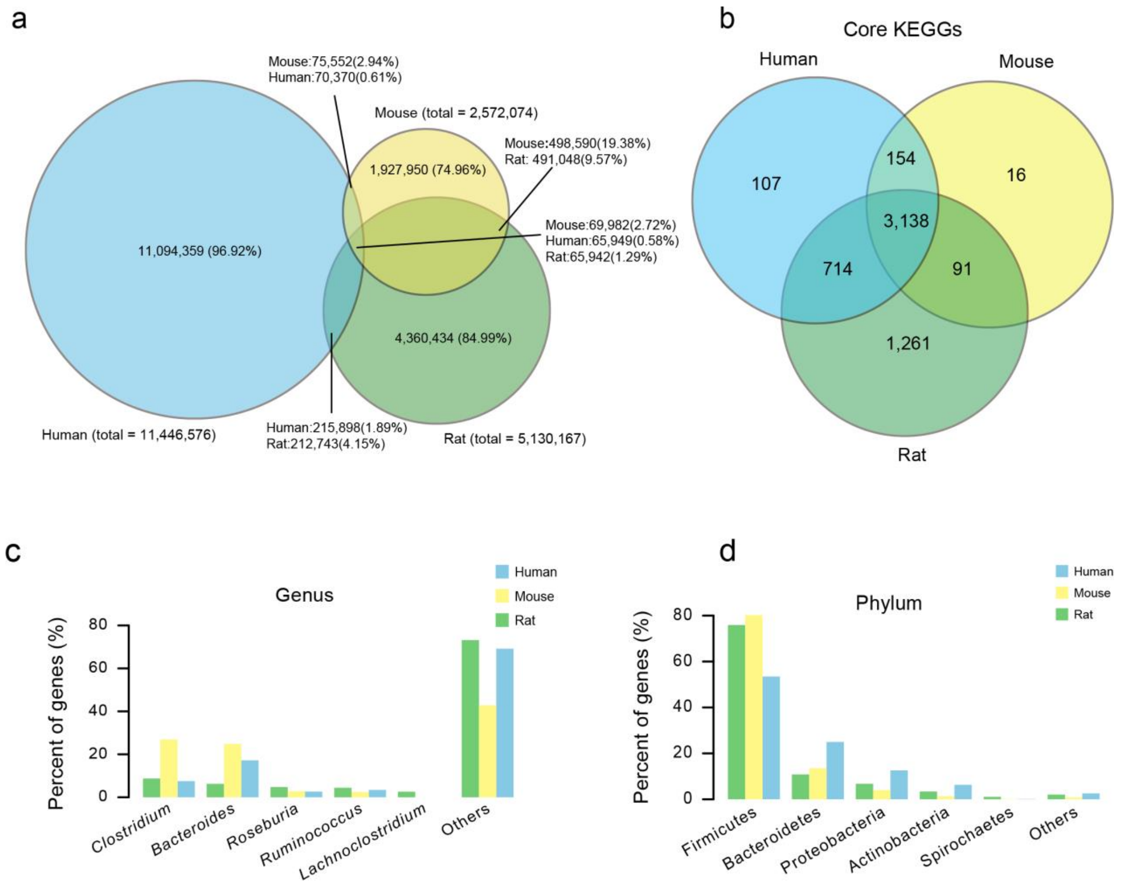

Figure 6. Comparison of the gut microbiome gene catalogs of human, mouse and rat. (a) Venn diagram of non-redundant genes shared between human (blue), mouse (yellow) and rat (green) gut microbiome catalogs. **(b)** Venn diagram of KO functions shared by the human, mouse and rat microbiota. **(c)** Percentage of genes in genera including *Clostridium*, *Bacteroides*, *Roseburia*, *Ruminococcus*, *Lachnoclostridium* in the gut microbial gene catalogs of rat, human and mouse.**(d)** The percentages of genes assigned to Fimicutes, Bacteroidetes, Proteobacteria, Aclinobacteria, Spirochaetes in the gut microbiomes of rat, human and mouse, respectively.

Conclusions

The newly established catalogue of the SD rat gut metagenome comprised ~5.1 million (M) non-redundant genes, which is almost twice the number of microbial gene in the mouse catalog comprising 2.6 M genes established be sequencing samples from different facilities and different mouse strain, and also including samples from low fat fed as well as high fat fed mice. Not

surprisingly, the overlap between microbial gene in rat and mouse is larger than between the rodents and human. However, the overall conclusion based on the available catalogs of gene in the gut microbiota of human[35], mouse[34], rat and pig [36] points to the remarkable differences in gene sequences in these four mammalian species implying that specific catalog for each mammalian species need to be produced for detailed analyses of the structural and functional analyses of the gut microbiota even though the microbiotas of the four mammals functionally are closely related. Thus, we envisage that the present catalog of genes in the rat gut microbiome will serve as a valuable resource for future work using rats as a model for investigating the role of the gut microbiota and the interactions with the host in health and disease.

Funding

This work was financially supported by grants from the Macau Technology Development Fund (102/2016/A3), the Shenzhen Municipal Government of China (JSGG20160229172752028, JCYJ20160229172757249) and the National Natural Science Foundation of China (Grant No. 81670606）.

Availability of supporting data

The sequencing reads from each sequencing library have been deposited at EBI with the accession number: PRJEB22973. The reference catalogue of the 5.1 million genes and related data in this article are available in the *GigaScience* database, GigaDB[37]. All supplementary figures and tables are provided as additional file.

Conflicts of interest

The authors declare no competing financial interests.

Author contributions

Design of the study, L. Liu. and H.J. Jia; Methodology, H.D. Pan, R.J. Guo, J. Zhu, Q. Wang, Y.M. Ju, Y.F. Zheng, Y. Xie, Z.Q. Liu, L.L. Lu, X.P. Li and T. Li; Investigation, H.D. Pan, Y.F. Zheng, R.Z. Li, and Z.W. Yuan; Data analysis, R.J. Guo, J. Zhu, Q. Wang, Y.M. Ju and L. Xiao; Sample collection, H.D. Pan, B. Tong; Writing of the first version of the manuscript:, H.D. Pan and R.J. Guo; Restructuring and extensive revision of the manuscript, KK. Supervision, of work, L. Liu and H.J. Jia; Funding acquisition, L. Liu and H.J. Jia.

Reference

1. Sommer, F., et al., *The resilience of the intestinal microbiota influences health and disease.* Nature reviews. Microbiology, 2017. **15**(10): p. 630-638.
2. Lynch, S.V. and O. Pedersen, *The Human Intestinal Microbiome in Health and Disease.* The New England journal of medicine, 2016. **375**(24): p. 2369-2379.
3. Quince, C., et al., *Shotgun metagenomics, from sampling to analysis.* Nature biotechnology, 2017. **35**(9): p. 833-844.
4. Liu, R., et al., *Gut microbiome and serum metabolome alterations in obesity and after weight-loss intervention.* Nature medicine, 2017. **23**(7): p. 859-868.
5. Zhang, X., et al., *The oral and gut microbiomes are perturbed in rheumatoid arthritis and partly normalized after treatment.* Nature medicine, 2015. **21**(8): p. 895-905.
6. Bhatt, A.P., M.R. Redinbo, and S.J. Bultman, *The role of the microbiome in cancer development and therapy.* CA: a cancer journal for clinicians, 2017. **67**(4): p. 326-344.
7. Pan, H., et al., *Whether Probiotic Supplementation Benefits Rheumatoid Arthritis*

Patients: A Systematic Review and Meta-Analysis. Engineering, 2017. **3**(1): p. 115-121.

8. Gu, Y., et al., *Analyses of gut microbiota and plasma bile acids enable stratification of patients for antidiabetic treatment.* Nature communications, 2017. **8**(1): p. 1785.

9. Vandeputte, D., et al., *Stool consistency is strongly associated with gut microbiota richness and composition, enterotypes and bacterial growth rates.* Gut, 2016. **65**(1): p. 57-62.

10. Johnson, C.L. and J. Versalovic, *The human microbiome and its potential importance to pediatrics.* Pediatrics, 2012. **129**(5): p. 950-60.

11. Lloyd-Price, J., G. Abu-Ali, and C. Huttenhower, *The healthy human microbiome.* Genome medicine, 2016. **8**(1): p. 51.

12. McInnes, I.B. and G. Schett, *The pathogenesis of rheumatoid arthritis.* The New England journal of medicine, 2011. **365**(23): p. 2205-19.

13. Waksman, B.H., C.M. Pearson, and J.T. Sharp, *Studies of arthritis and other lesions induced in rats by injection of mycobacterial adjuvant. II. Evidence that the disease is a disseminated immunologic response to exogenous antigen.* Journal of immunology, 1960. **85**: p. 403-17.

14. Pearson, C.M. and F.D. Wood, *Studies of arthritis and other lesions induced in rats by the injection of mycobacterial adjuvant. VII. Pathologic details of the arthritis and spondylitis.* The American journal of pathology, 1963. **42**: p. 73-95.

15. Cai, X., et al., *Manipulation of the induction of adjuvant arthritis in Sprague-Dawley rats.* Inflammation research : official journal of the European Histamine Research

Society ... [et al.], 2006. **55**(9): p. 368-77.

16. Hegen, M., et al., *Utility of animal models for identification of potential therapeutics for rheumatoid arthritis.* Annals of the rheumatic diseases, 2008. **67**(11): p. 1505-15.

17. Srinivasan, K., et al., *Combination of high-fat diet-fed and low-dose streptozotocin-treated rat: a model for type 2 diabetes and pharmacological screening.* Pharmacological research, 2005. **52**(4): p. 313-20.

18. Levin, B.E., et al., *Selective breeding for diet-induced obesity and resistance in Sprague-Dawley rats.* The American journal of physiology, 1997. **273**(2 Pt 2): p. R725-30.

19. Zhang, R. and L. Lao, *A new rat model of bone cancer pain.* Methods in molecular biology, 2012. **851**: p. 261-73.

20. Delbosc, S., et al., *Involvement of oxidative stress and NADPH oxidase activation in the development of cardiovascular complications in a model of insulin resistance, the fructose-fed rat.* Atherosclerosis, 2005. **179**(1): p. 43-9.

21. Flemer, B., et al., *Fecal microbiota variation across the lifespan of the healthy laboratory rat.* Gut microbes, 2017. **8**(5): p. 428-439.

22. Cronstein, B.N., *Low-dose methotrexate: a mainstay in the treatment of rheumatoid arthritis.* Pharmacological reviews, 2005. **57**(2): p. 163-72.

23. Wu, J., et al., *Simultaneous Determination of Multiple Components in Guanjiekang in Rat Plasma via the UPLC-MS/MS Method and Its Application in Pharmacokinetic Study.* Molecules, 2016. **21**(12).

24. Chen, X.M., et al., *Systemic Review and Meta-Analysis of the Clinical Efficacy and*

Adverse Effects of Zhengqing Fengtongning Combined with Methotrexate in Rheumatoid Arthritis. Evidence-based complementary and alternative medicine : eCAM, 2015. **2015**: p. 910376.

25. Qin, J., et al., *A metagenome-wide association study of gut microbiota in type 2 diabetes.* Nature, 2012. **490**(7418): p. 55-60.
26. Huang, J., et al., *A reference human genome dataset of the BGISEQ-500 sequencer.* GigaScience, 2017. **6**(5): p. 1-9.
27. Mende, D.R., et al., *Assessment of metagenomic assembly using simulated next generation sequencing data.* PloS one, 2012. **7**(2): p. e31386.
28. Kultima, J.R., et al., *MOCAT: a metagenomics assembly and gene prediction toolkit.* PloS one, 2012. **7**(10): p. e47656.
29. Li, R., et al., *SOAP2: an improved ultrafast tool for short read alignment.* Bioinformatics, 2009. **25**(15): p. 1966-7.
30. Peng, Y., et al., *IDBA-UD: a de novo assembler for single-cell and metagenomic sequencing data with highly uneven depth.* Bioinformatics, 2012. **28**(11): p. 1420-8.
31. Hyatt, D., et al., *Prodigal: prokaryotic gene recognition and translation initiation site identification.* BMC bioinformatics, 2010. **11**: p. 119.
32. Li, W. and A. Godzik, *Cd-hit: a fast program for clustering and comparing large sets of protein or nucleotide sequences.* Bioinformatics, 2006. **22**(13): p. 1658-9.
33. Xie, H., et al., *Shotgun Metagenomics of 250 Adult Twins Reveals Genetic and Environmental Impacts on the Gut Microbiome.* Cell systems, 2016. **3**(6): p. 572-584 e3.

34. Xiao, L., et al., *A catalog of the mouse gut metagenome.* Nature biotechnology, 2015. **33**(10): p. 1103-8.

35. Li, J., et al., *An integrated catalog of reference genes in the human gut microbiome.* Nature biotechnology, 2014. **32**(8): p. 834-41.

36. Xiao, L., et al., *A reference gene catalogue of the pig gut microbiome.* Nature microbiology, 2016: p. 16161.

37. Pan H, Guo R, Zhu J, Wang Q, Ju Y, Xie Y, et al.: A gene catalogue of the Sprague-Dawley rat gut metagenome GigaScience Database 2018. http://dx.doi.org/10.5524/100440

Supplementary Figure

Click here to access/download
Supplementary Material
Supllementary Figure.docx

Click here to access/download
Supplementary Material
sTable1. The body weight of rats.xlsx

Click here to access/download
Supplementary Material
sTable2.Data for Gene catalogue.xlsx

Click here to access/download
Supplementary Material
sTable3.Reads of SE100 for Alignment.xlsx

Click here to access/download
Supplementary Material
New-sTab4.Gene distribution in pathways.20180315.xlsx

Supplementary Table

Click here to access/download
Supplementary Material
New-sTab5.Gene distribution in modules.20180315.xlsx

Supplementary Table

Click here to access/download
Supplementary Material
sTable6.Mapping to Catalogues.xlsx

Response to reviewers (doc)

Click here to access/download

Supplementary Material

2018-03-22 Responses to Gigascience.docx

Response to reviews (PDF)

Click here to access/download
Supplementary Material
2018-03-22 Responses to Gigascience.pdf
