# Supplementary material for: A gene catalogue of the Sprague-Dawley rat gut metagenome
Source: Gigascience. 2018 May 11;7(5):giy055. doi: 10.1093/gigascience/giy055 (PMC5967468; doi:10.1093/gigascience/giy055)
Supplement: Supplemental material [file giy055_supp.zip › 2018-03-22 Responses to Gigascience.pdf]

Editorial Office

<u>Responses to "GIGA-D-17-00275"</u>

Thank you for your kind letter of December 8th, 2017. Based on your comment and request, we have made extensive modification on the original manuscript. We hereby send you the revised clean manuscript in the formats of both a PDF file and a MS word file for your consideration. We also include the revised manuscript with all the changes marked in red for easier comparison and editing purposes.

Below follow our responses to the comments and suggestions made by the reviewers.

Responses to Reviewer #1:

<u>General comment:</u> *The data described in this manuscript presents many levels of genetic information from rat feces. It will likely be used as a reference by researchers working in gut function and characterization, and is therefore a valuable contribution to the scientific community. Generally, I find that the manuscript needs English language editing and careful proofreading to weed out small irregularities (e.g. number inconsistencies? on p1.l12) and to make each section more concise (for example, but not only, the section on germ free animals which is not directly related to the present dataset). The experimental and bioinformatical procedure, including sample handling, DNA extraction, assembly, gene prediction, taxonomic assignment, and gene functional annotation is sound, and the descriptions are sufficient.*

<u>Response:</u> Thanks for your valuable suggestions. We have completely restructured the background section, and hope that it now appears easier to follow and relevant.

<u>Specific comments:</u>

1. Background, l40: I believe religious considerations are covered by ethics considerations and do not need to be mentioned.

<u>Response:</u> We completely agree, and the section has been completely rewritten.

2. DNA extraction, l26: the sentence "The standard protocol as described in ref, including DNA fragmentation and selection, end repair and a-tailing, and circularization" is not clear.

Response: We have revised the section, so it now reads "We constructed sequencing library following the BGISEQ-500 instruction and using the standard protocol with minor modification. In brief, the genomic DNA was fragmented and DNA fragments between 100 base pairs (bp) and ~300 bp were selected. The selected DNA fragments were repaired and modified. A dTTP tailed adapter sequence was ligated to both ends of the DNA fragments and the fragments were further amplified and subjected single-strand circularization." (line 129-133 in the revised manuscript)

3. Data preprocess, l56: "quality value less than 3...? which quality measure? Phred-like?

Response: We used Phred quality score. The Section has been revised for clarification; it now reads "To remove or trim low quality reads we used our in-house Perl script and the quality was assessed by Phred quality score." (line 140 in the revised manuscript)

4. Figure 5; include description of what the modules and pathways consist of somewhere in the text

Response: The compositions of the modules and pathways are now described in the text (line 214-217 in the revised manuscript). The section now reads "Among these, we noted metabolic functions including pathways or modules involved in carbohydrates, amino acid, and energy metabolism; environmental information processing including membrane transport pathways or modules and genetic information processing including replication and repair, translation and transcription (Table S4, S5) ."

5. Comparison of human, mouse, rat gene catalogue, l9: "...%of the reads were allowed for mapping to?. Not understandable

Response: The section has been revised. It now reads “An average of 20.45% and 25.41% of the reads of the SD rats mapped to the non-redundant gene sets of the mouse and human gut microbiome, respectively (Table S6)” (line 238-239 in the revised manuscript).

6. A discussion of the use of fecal samples to evaluate the gut microbiota should be included somewhere.

Response: We added a section discussing this aspect in the Background section (Lines 47-52).

7. A detailed description of the work carried out by each of the 28 authors should also be included, particularly as this is a very high number of authors for a Data Note of limited size and complexity.

Response: We have added a detailed description of the work (line281-287 in the revised manuscript) and removed some authors with fewer contributions. In addition Professor Karsten Kristiansen critically and extensively revised and modified the manuscript, and he is now included a co-author. The final list of authors is shown below:

Hudan Pan[1,10], Ruijin Guo[1,2,3,10], Jie Zhu[2,3,10], Qi Wang[2,3,6], Yanmei Ju[2,3], Ying Xie[1], Yanfang Zheng[1,5], Zhifeng Wang[2,3], Ting Li[1], Zhongqiu Liu[4], Linlin Lu[4], Fei Li[2,3,6], Bin Tong[2,3], Liang Xiao[2,3,7], Xun Xu[2,3]Runze Li[1], Zhongwen Yuan[1],Huanming Yang[2,3], Jian Wang[2,3] , Karsten Kristiansen[2,3,9], Huijue Jia[1,2,3,8] & Liang Liu[1]

Response to Reviewer #2:

General comment: *In this manuscript, Pan and colleagues generated a gene set of Sprague-Dawley (SD) rat gut metagenome using 98 stool samples from 49 rats in 7 groups at 2 time-points. The reported set has ~ 5 million non-redundant genes and ~70% of the reads can be aligned to it. The rat gut metagenome catalogue was then compared to that of the mouse and the human gut metagenome catalogues at the phylum and genus levels. This is a useful resource and is of interest to many researchers but I have the following concerns:*

Major concerns:

1) Why each sample was assembled alone? No justification is given for this approach. Would assembling all the samples at once produce better assembly (N50, number of genes, etc ..)?

Response: The 98 fecal samples, which produced high-quality reads for assembly, were collected from 49 SD rats at 2 time-points before and after treatment in 7 groups (as shown in Fig 1). A high sequence complexity was expected between pair-wised samples and across all groups. De novo assembly of each sample reduces assembly errors compared to a co-assembling strategy.

2) The authors use the following k-mers 27, 37, 47, 57, 67, 77, 87 and 97 for their assembly. Did the authors run optimizing trials and found those k-mers give the best assembly? Given that their paired-end reads are 50 bases, only three k-mers will be used for PE reads. Would using a lower "--mink" value produce better assembly?

Response: We apologize for this confusion. Actually, two types of reads, 100bp single-end reads and 50bp paired-end reads, were pooled to assemble ilonger contigs using parameter '-r pe.fa -l se.fa'(line134-137 in the revised manuscript). A basic feature of IDBA-UD is the multi k-mer assembly approach which iterates k-mer values in our study from "--mink 27" to "--maxk 97 " by "--step 10" in order to stepwise improve the De-Bruijn graph and the resulting assembly. To balance the

number and accuracy of these assemblies, we adopted this series of k-mer values based on experience that the closest odd number larger or equal to half the average read length should be used.

3) Why pre-correction was not used in IDBA-UD assembly although it is used by IDBA-UD developer for metagenome assembly? Would including "--pre_correction" in IDBA-UD enhances the assembly?

<u>Response:</u> We'd like to thank the Referee for this suggestion. We have now chose reads of 10 samples based on their N50 (5 samples from top rank and 5 samples from bottom rank) and re-assembled them independently by IDBA-UD with parameter "--pre_correction". However, likely due to the sequencing depths our data is not extremely uneven, and we did not observe a significant difference in assembled indices. Please, consult the figure below.

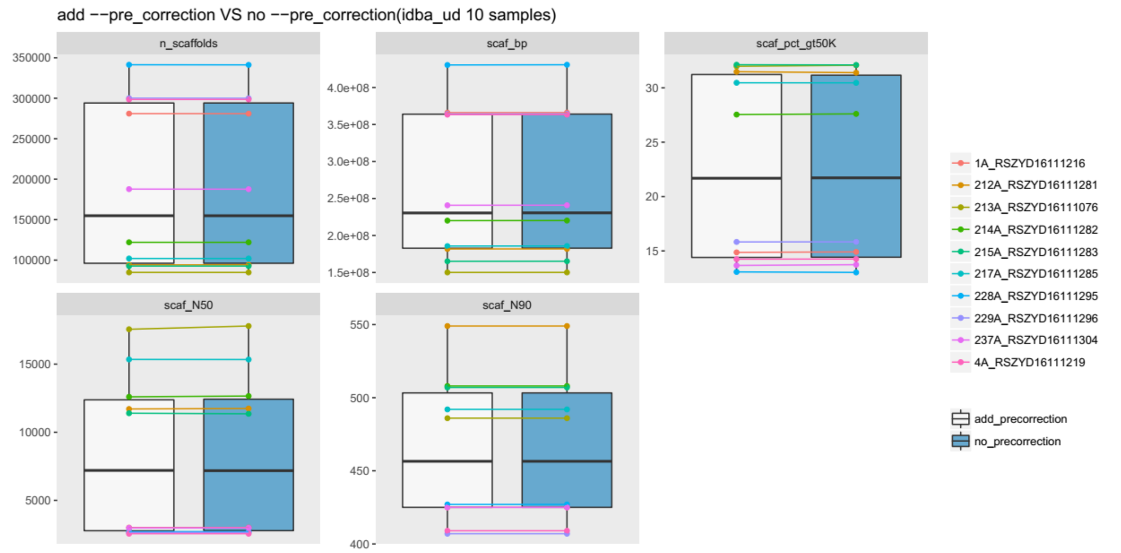

Figure 1 Comparison of "—pre-correction" and "—no pre-correction" in IDBA-UD.

4) A PCA analysis is needed. It is important to know how the samples cluster based on gene counts and taxa counts.

Response: We would like to thank the Referee for this suggestion and we have added a new figure (see below) in the supplementary material as Fig.S1. The text (line174-176), now reads "When accounting for the samples cluster based on gene counts and genus counts in the seven groups, a principal coordinates analysis (PCoA) of the abundance profiles at the level of gene or genera could not clearly separate the gut microbiome in the groups, except for the high fat diet group (**Fig. S1**)."

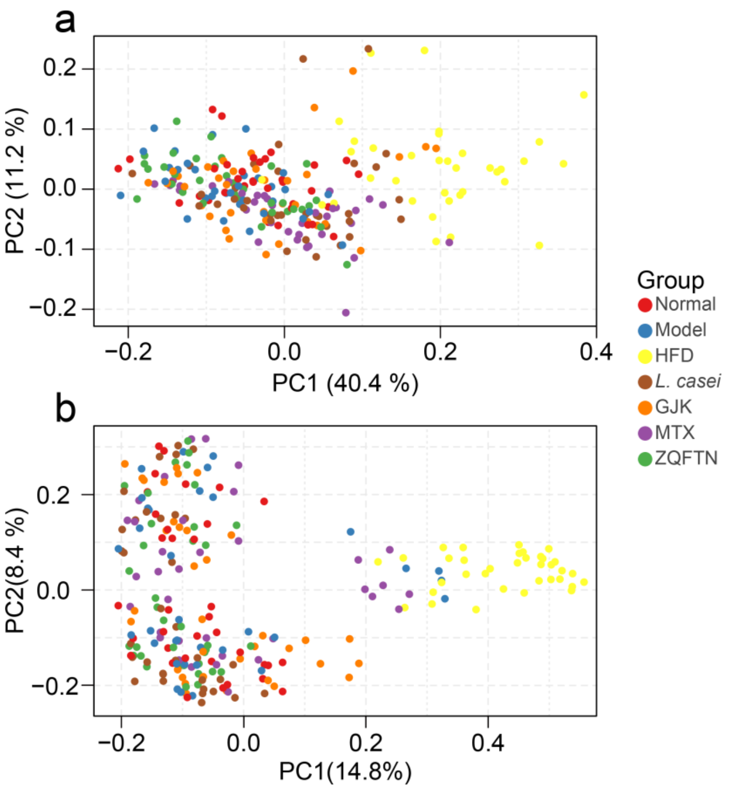

Figure 2 A PCoA of the 98 samples of the 7 groups at the gene (a) and the genus (b) levels.

5) Approximate 35% of the genes can't be assigned to any phyla and 47% of the genes can't be assigned to KEGG KOs. How many of those genes overlap (can't be assigned to phyla and can't be assigned to KOs)? Are those misassembled genes?

Response: In our study, we found that approximate 35% of the genes cannot be assigned to any phyla and 47% of the genes cannot be assigned to KEGG KOs. This is comparable to what has been observed for other published gut bacterial gene catalogsIn the rat catalog 1,380,083 genes cannot be assigned to either a KO or a

phylum (shown in below). According to early studies which include Sanger sequencing, the misassembly rate was only 0.014 per kb (SOAPdenovo; Qin et al. 2010, Nature.). And by removing redundancy of genes from difference samples (CD-HIT, 95% identity, 90% overlap) com, the potential contribution from misassembly is further reduced. The relative high proportion of genes that cannot be assigned to any phyla or KEGG KOs probably relates to the incomplete coverage of gut microbial genes in the current reference genomes.

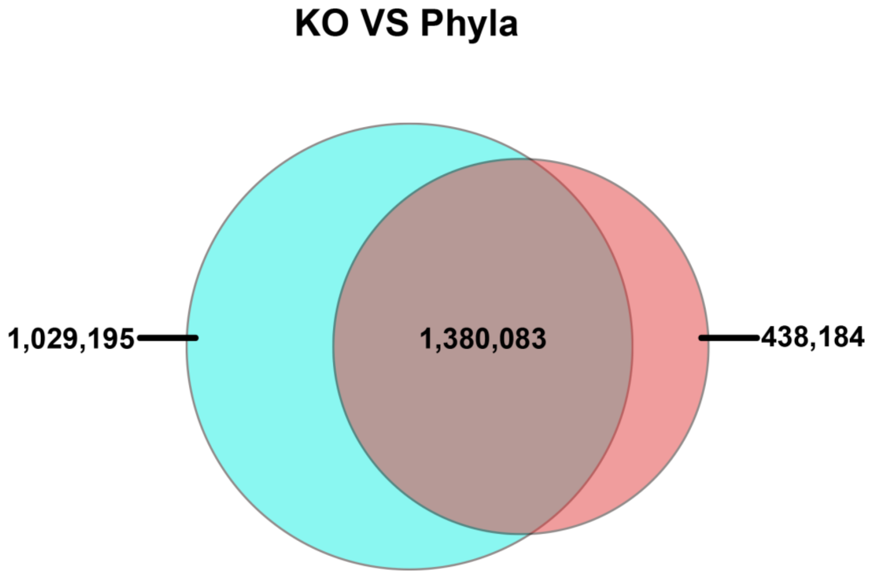

Figure 3 Genes that cannot be assigned to a phylum or a KO.

6) A comparison between the rat, mouse and human metagenome genes at the functional level using KEGG KOs, pathways and modules is needed.

Response: We have now included a comparison between the rat, mouse and human metagenome genes at the functional level using KEGG KOs as shown below and in the text Fig.6b.

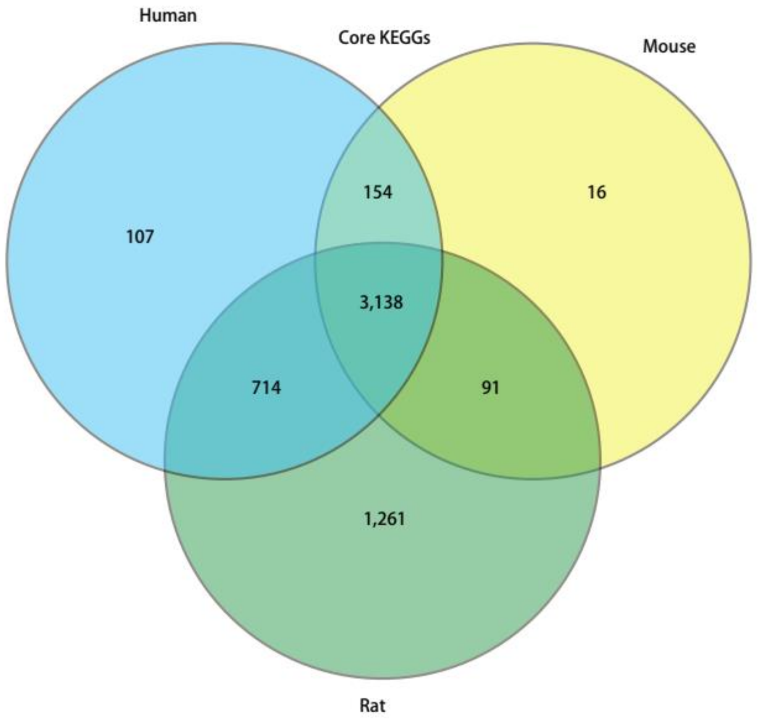

Figure 4 Venn diagram of score KEGGs shared between the human (blue), mouse (yellow) and rat (green) gut microbiome catalogs.

7) What percentage of genes (not reads) overlap between rat, mouse and human?

Response: We have conducted an analysis determining the of overlap genes (not reads) between rat, mouse and human as shown below and in the text Fig.6a.

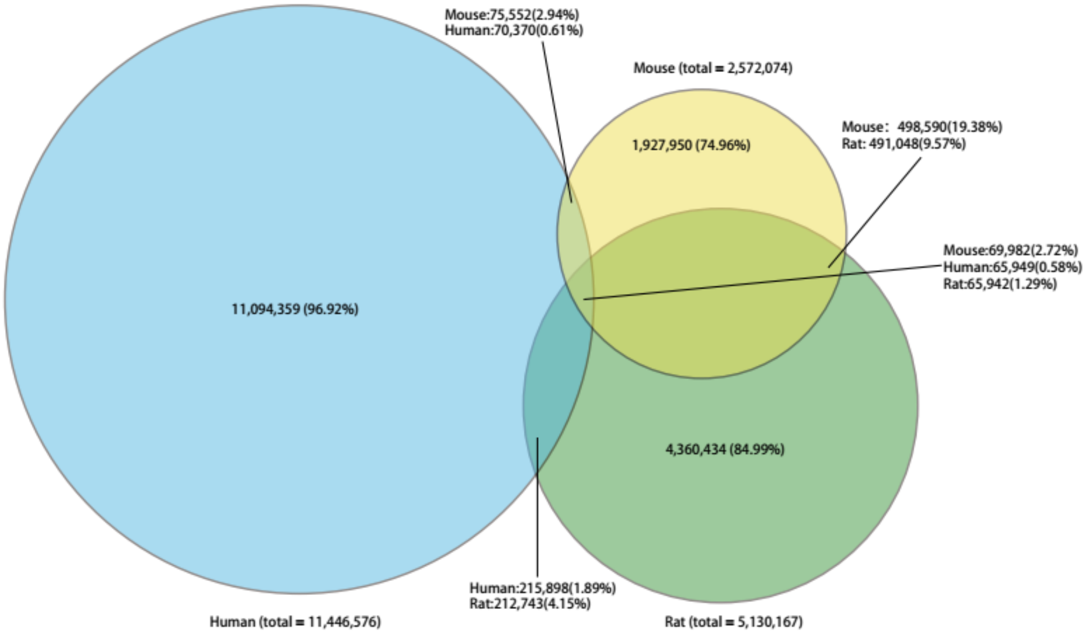

Figure 5 Venn diagram of genes shared by the human, mouse and rat catalogs.

8) The reported comparison with human metagenome gene catalogue uses a recent twin study. A comparison using the Human Microbiome project (HMP) gene catalogue is needed since the HMP is the golden standard in the field.

Response: The gene set identified in TwinsUK cohort were merged with the Integrated reference gene catalogue (ICG), leading to an updated gene catalogue containing 11.4 million genes from 1,517 fecal samples of 1,320 people around the world. We believe it is a more comprehensive resource for metagenomics studies on humans. So in this study, we used the human metagenome gene catalogue in as reported in the TwinsUK cohort rather than the Human Microbiome project (HMP) gene catalog.

Minor concerns:

1) Figure 2: figure colors don't match the description given in figure legend. Human in the legend is red but shown as light blue in the figure.

Response: We have revised the description in figure legend (line 179 in the revised manuscript). Thanks for the comment.

2) Figure 5 legend, change white to yellow in: "Yellow: the area of white reflects unknown function annotation"

Response: We have revised the figure legend (line 205 in the revised manuscript).

3) Include a table linking EBI sample IDs to sample IDs shown in supplemental data.

Response: We have added a table linking EBI sample IDs shown in supplemental data. Thanks for your suggestion.

4) Define MTX, GJK and ZQFTN.

Response: We have described MTX, GJK and ZQFTN in the revised manuscript (line 96-99 in the revised manuscript). The section now reads "MTX is a widely used disease-modifying anti-rheumatic drug. GJK is a Chinese experimental herb formula and ZQFTN is a monomer drug derived from the Chinese traditional herb-*Caulis Sinomenii*. These three drugs have been used in China for RA therapy for a long time with good effectiveness."

5) There are many typos in the manuscript that need to be addressed, for example, "that" in: "It is still unknown that how colonies of microbiota are established and changed". A carful round of editing is needed.

Response: The manuscript has been extensively modified and revised.

We hope the above our responses and the revised manuscript address the comments

and suggestions from the reviewers and we hope that the revised manuscript now is acceptable for publication in GigaScience. We are looking forward to hearing from you.

Yours sincerely,

Prof. Liang Liu, MD., Ph.D.

President and Chair Professor of Macau University of Science and Technology,

Director of the State Key Laboratory of Quality Research in Chinese Medicine,

Macau University of Science and Technology

Taipa, Macau, China.

Tel: +853-8897 2238

Fax: + 853-2882 3312

E-mail Address: lliu@must.edu.mo
