# Supplementary figures and images for: A gene catalogue of the Sprague-Dawley rat gut metagenome
Source: Gigascience. 2018 May 11;7(5):giy055. doi: 10.1093/gigascience/giy055 (PMC5967468; doi:10.1093/gigascience/giy055)

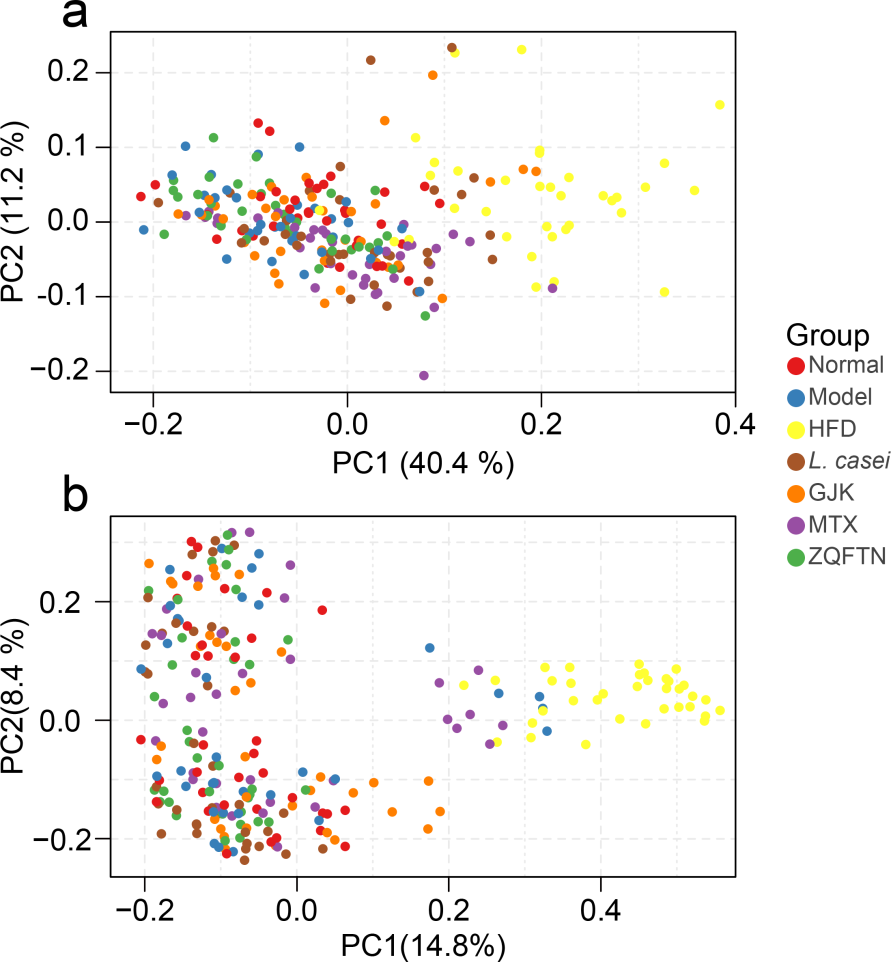


Supplementary Figure 1. A PCoA analysis of the 98 samples in 7 groups in gene (a) and genus (b) levels.

Supplement: Supplemental material [file giy055_supp.zip › Supllementary Figure.docx]
