# Supplementary material for: A gene catalogue of the Sprague-Dawley rat gut metagenome
Source: Gigascience. 2018 May 11;7(5):giy055. doi: 10.1093/gigascience/giy055 (PMC5967468; doi:10.1093/gigascience/giy055)
Supplement: Reviewer_1_Report_(Original_Submission) -- Tue Sparholt Jørgensen — 12/5/2017 Reviewed [file giy055_reviewer_1_report_(original_submission).pdf]

Reviewer Report

Title: A gene catalogue of the Sprague-Dawley rat gut metagenome

Version: Original Submission **Date:** 12/5/2017

Reviewer name: Tue Sparholt Jørgensen

Reviewer Comments to Author:

The data described in this manuscript presents many levels of genetic information from rat feces. It will likely be used as a reference by researchers working in gut function and characterization, and is therefore a valuable contribution to the scientific community. Generally, I find that the manuscript needs English language editing and careful proofreading to weed out small irregularities (e.g. number inconsistencies? on p1.l12) and to make each section more concise (for example, but not only, the section on germ free animals which is not directly related to the present dataset). The experimental and bioinformatical procedure, including sample handling, DNA extraction, assembly, gene prediction, taxonomic assignment, and gene functional annotation is sound, and the descriptions are sufficient. Specific comments: Background, l40: I believe religious considerations are covered by ethics considerations and do not need to be mentioned.DNA extraction, l26: the sentence "The standard protocol as described in ref, including DNA fragmentation and selection, end repair and a-tailing, and circularization" is not clear. Data preprocess, l56: "quality value less than 3..." which quality measure? Phred-like?Figure 5; include description of what the modules and pathways consist of somewhere in the text Comparison of human, mouse, rat gene catalogue, l9: "...%of the reads were allowed for mapping to ...". Not understandableA discussion of the use of fecal samples to evaluate the gut microbiota should be included somewhere.A detailed description of the work carried out by each of the 28 authors should also be included, particularly as this is a very high number of authors for a Data Note of limited size and complexity.

Level of Interest

Please indicate how interesting you found the manuscript: An article whose findings are important to those with closely related research interests

Quality of Written English

Please indicate the quality of language in the manuscript: Not suitable for publication unless extensively edited

Declaration of Competing Interests

Please complete a declaration of competing interests, considering the following questions:

- Have you in the past five years received reimbursements, fees, funding, or salary from an organisation that may in any way gain or lose financially from the publication of this manuscript, either now or in the future?
- Do you hold any stocks or shares in an organisation that may in any way gain or lose financially from the publication of this manuscript, either now or in the future?

- Do you hold or are you currently applying for any patents relating to the content of the manuscript?
- Have you received reimbursements, fees, funding, or salary from an organization that holds or has applied for patents relating to the content of the manuscript?
- Do you have any other financial competing interests?
- Do you have any non-financial competing interests in relation to this paper?

If you can answer no to all of the above, write 'I declare that I have no competing interests' below. If your reply is yes to any, please give details below.

I declare that I have no competing interests

I agree to the open peer review policy of the journal. I understand that my name will be included on my report to the authors and, if the manuscript is accepted for publication, my named report including any attachments I upload will be posted on the website along with the authors' responses. I agree for my report to be made available under an Open Access Creative Commons CC-BY license (http://creativecommons.org/licenses/by/4.0/). I understand that any comments which I do not wish to be included in my named report can be included as confidential comments to the editors, which will not be published.

I agree to the open peer review policy of the journal

To further support our reviewers, we have joined with Publons, where you can gain additional credit to further highlight your hard work (see: https://publons.com/journal/530/gigascience). On publication of this paper, your review will be automatically added to Publons, you can then choose whether or not to claim your Publons credit. I understand this statement.

Yes
