# Supplementary material for: A gene catalogue of the Sprague-Dawley rat gut metagenome
Source: Gigascience. 2018 May 11;7(5):giy055. doi: 10.1093/gigascience/giy055 (PMC5967468; doi:10.1093/gigascience/giy055)
Supplement: Reviewer_2_Report_(Original_Submission) -- Raad Gharaibeh — 12/7/2017 Reviewed [file giy055_reviewer_2_report_(original_submission).pdf]

Reviewer Report

Title: A gene catalogue of the Sprague-Dawley rat gut metagenome

Version: Original Submission **Date:** 12/7/2017

Reviewer name: Raad Gharaibeh

Reviewer Comments to Author:

In this manuscript, Pan and colleagues generated a gene set of Sprague-Dawley (SD) rat gut metagenome using 98 stool samples from 49 rats in 7 groups at 2 time-points. The reported set has ~ 5 million non-redundant genes and ~70% of the reads can be aligned to it. The rat gut metagenome catalogue was then compared to that of the mouse and the human gut metagenome catalogues at the phylum and genus levels. This is a useful resource and is of interest to many researchers but I have the following concerns:Major concerns:1) Why each sample was assembled alone? No justification is given for this approach. Would assembling all the samples at once produce better assembly (N50, number of genes, etc ..)?2) The authors use the following k-mers 27, 37, 47, 57, 67, 77, 87 and 97 for their assembly. Did the authors run optimizing trials and found those k-mers give the best assembly? Given that their paired-end reads are 50 bases, only three k-mers will be used for PE reads. Would using a lower "--mink" value produce better assembly? 3) Why pre-correction was not used in IDBA-UD assembly although it is used by IDBA-UD developer for metagenome assembly? Would including "--pre_correction" in IDBA-UD enhances the assembly?4) A PCA analysis is needed. It is important to know how the samples cluster based on gene counts and taxa counts.5) Approximate 35% of the genes can't be assigned to any phyla and 47% of the genes can't be assigned to KEGG KOs. How many of those genes overlap (can't be assigned to phyla and can't be assigned to KOs)? Are those misassembled genes?6) A comparison between the rat, mouse and human metagenome genes at the functional level using KEGG KOs, pathways and modules is needed. 7) What percentage of genes (not reads) overlap between rat, mouse and human?8) The reported comparison with human metagenome gene catalogue uses a recent twin study. A comparison using the Human Microbiome project (HMP) gene catalogue is needed since the HMP is the golden standard in the field.Minor concerns:1) Figure 2: figure colors don't match the description given in figure legend. Human in the legend is red but shown as light blue in the figure.2) Figure 5 legend, change white to yellow in: "Yellow: the area of white reflects unknown function annotation" 3) Include a table linking EBI sample IDs to sample IDs shown in supplemental data.4) Define MTX, GJK and ZQFTN.5) There are many typos in the manuscript that need to be addressed, for example, "that" in: "It is still unknown that how colonies of microbiota are established and changed". A carful round of editing is needed.

Level of Interest

Please indicate how interesting you found the manuscript: An article of importance in its field

Quality of Written English

Please indicate the quality of language in the manuscript: Needs some language corrections before being published

Declaration of Competing Interests

Please complete a declaration of competing interests, considering the following questions:

- Have you in the past five years received reimbursements, fees, funding, or salary from an organisation that may in any way gain or lose financially from the publication of this manuscript, either now or in the future?
- Do you hold any stocks or shares in an organisation that may in any way gain or lose financially from the publication of this manuscript, either now or in the future?
- Do you hold or are you currently applying for any patents relating to the content of the manuscript?
- Have you received reimbursements, fees, funding, or salary from an organization that holds or has applied for patents relating to the content of the manuscript?
- Do you have any other financial competing interests?
- Do you have any non-financial competing interests in relation to this paper?

If you can answer no to all of the above, write 'I declare that I have no competing interests' below. If your reply is yes to any, please give details below.

I declare that I have no competing interests

I agree to the open peer review policy of the journal. I understand that my name will be included on my report to the authors and, if the manuscript is accepted for publication, my named report including any attachments I upload will be posted on the website along with the authors' responses. I agree for my report to be made available under an Open Access Creative Commons CC-BY license (http://creativecommons.org/licenses/by/4.0/). I understand that any comments which I do not wish to be included in my named report can be included as confidential comments to the editors, which will not be published.

I agree to the open peer review policy of the journal

To further support our reviewers, we have joined with Publons, where you can gain additional credit to further highlight your hard work (see: https://publons.com/journal/530/gigascience). On publication of this paper, your review will be automatically added to Publons, you can then choose whether or not to claim your Publons credit. I understand this statement.

Yes
